# Creating genetic reports that are understood by nonspecialists: a case study

**DOI:** 10.1038/s41436-019-0649-0

**Published:** 2019-09-11

**Authors:** Gabriel Recchia, Antonia Chiappi, Gemma Chandratillake, Lucy Raymond, Alexandra L. J. Freeman

**Affiliations:** 1 Winton Centre for Risk and Evidence Communication, University of Cambridge, Cambridge, UK; 2 Institute of Continuing Education, University of Cambridge, Cambridge, UK; 3 East of England NHS Genomic Medicine Centre, London, UK; 4 Department of Medical Genetics, University of Cambridge, Cambridge, UK; 5 NIHR Bioresource-Rare Disease, London, UK

**Keywords:** genetic test reports, comprehension, user-centered design, risk communication, personalized medicine

## Abstract

**Purpose:**

Guidelines recommend that genetic reports should be clear to nonspecialists, including patients. We investigated the feasibility of creating reports for cystic fibrosis carrier testing through a rapid user-centered design process that built on a previously developed generic template. We evaluated the new reports’ communication efficacy and effects on comprehension against comparable reports used in current clinical practice.

**Methods:**

Thirty participants took part in three rounds of interviews. Usability problems were identified and rectified in each round. One hundred ninety-three participants took part in an evaluation of the resulting reports measuring subjective comprehension, risk probability comprehension, perceived communication efficacy, and other factors, as compared with standard reports.

**Results:**

Participants viewing the user-centered reports rated them as clearer, easier to understand, and more effective at communicating key information than standard reports. Both groups ended up with equivalent knowledge of risk probabilities, although we observed differences in how those probabilities were perceived.

**Conclusion:**

Our findings demonstrate that by starting with a patient-friendly generic report template and modifying it for specific scenarios with a rapid user-centered design process, reports can be produced that are more effective at communicating key information. The resulting reports are now being implemented into clinical care.

## INTRODUCTION

Genetic and genomic testing is becoming increasingly widely available due to falling costs of testing, new referral pathways, increased integration of such testing into mainstream clinical practice, and other initiatives such as the National Health Service (NHS) Long Term Plan and the Improving Outcomes through Personalised Medicine effort in the United Kingdom.^1^ As access to such services expands, nonspecialist clinicians are increasingly tasked with explaining the results of these tests to patients. In some cases, patients may be faced with the prospect of interpreting reports themselves without guidance. For example, patients in some countries can obtain copies of their test results directly from testing laboratories.^2^

Research suggests that even clinicians have difficulties understanding genetic reports,^3,4^ and many researchers have recognized the need for clearer reports in light of variability among individuals in numeracy, health literacy, and genetic literacy.^2,5,6^ Guidelines state that reports should be clear and comprehensible to nonspecialists, and provide some guidance on how to achieve this.^2,7–14^ Despite widespread adoption of some guidelines, such as those of the American College of Medical Genetics and Genomics (ACMG),^7^ studies investigating patients’ and nonspecialists’ satisfaction and perceptions find that existing reports leave substantial room for improvement.^4,15–17^ Genomic reports are especially challenging due to lack of standardization^18,19^ and the complexity and uncertainty of the information involved.^20^

There have been attempts to make the interpretation of laboratory reports clearer to nonspecialist clinicians,^16,21–25^ but far fewer to make them clearer to patients. In 2014, Haga et al.^2^ noted that “only one study has described efforts to develop a patient-friendly pathology report” (p. 4). There have since been some efforts to make genetic or genomic test reports more patient-friendly,^2,14,26–30^ including in the direct-to-consumer (DTC) industry.^5,30^ However, work of this kind still appears rarely outside the DTC space, and there has been little published (or made publicly available) about the development of DTC reports. There are therefore few examples to guide the design and evaluation of a patient-friendly genetic report.

In industry, it is common for new products to be developed via a user-centered design^31,32^ approach whereby changes are made in an iterative process, taking into account the context in which the product will be used, key requirements, and feedback from users. Typically, multiple rounds of evaluation are conducted, monitoring metric(s) of interest (e.g., number and severity of usability issues, time required for users to accomplish a task, etc.) to assess what changes are needed. The iterative process continues until some stopping criterion is reached.

With rare exceptions,^25,28^ user-centered design is not generally used as a guiding framework in the context of noncommercial genetic report development. Our aim was to determine whether such a process could be used to efficiently produce genetic report templates suitable for implementation. If such reports could be shown to communicate more effectively to laypersons, this would suggest a reasonable, cost-efficient approach that could be emulated by others.

Our approach was to split the design phase into two. In a first stage, patients, nonspecialist clinicians and genetic testing experts participated in the development of a report template for a fictional genetic condition. This work (submitted for publication) resulted in a generic template that could be adapted to specific use cases. We chose cystic fibrosis (CF) carrier testing as our specific use case as primary care physicians were being directed to order CF tests (and hence receive and communicate results) in our local health-care region. There was therefore a need to ensure that reports from such testing were clear to nonspecialist readers. Our study provides preliminary findings regarding benefits and limits of what can be expected from a design process of this kind.

## MATERIALS AND METHODS

One design feature of the generic template was to accommodate the needs of both genetic specialists and nonspecialists (including patients) by separating sections containing technical information from those in “plain English.” Therefore, our reports had both a “patient-centered” page and a “clinician-centered” page, with the second page intended for health professionals.

Five two-page draft reports were developed representing common scenarios for CF carrier testing, where the reasons for referral were the following: partner heterozygous for p.Phe508del (positive and negative); familial p.Phe508del (positive and negative); and family history (unknown variant), negative report only. Reasons for referral were stated in simpler language on the patient-centered page of each report. Our initial reports were developed on the basis of our previously designed generic report template, with input from members of a working group who produced recommendations based on a revision of the Association for Clinical Genomic Science general reporting guidelines. This group included members of the Regional NHS Clinical Genetics Laboratory in Cambridge, clinical geneticists, genetic counselors, National External Quality Assessment Service members, and other experts in the reporting of genetic test results.

**Fig. 1:**
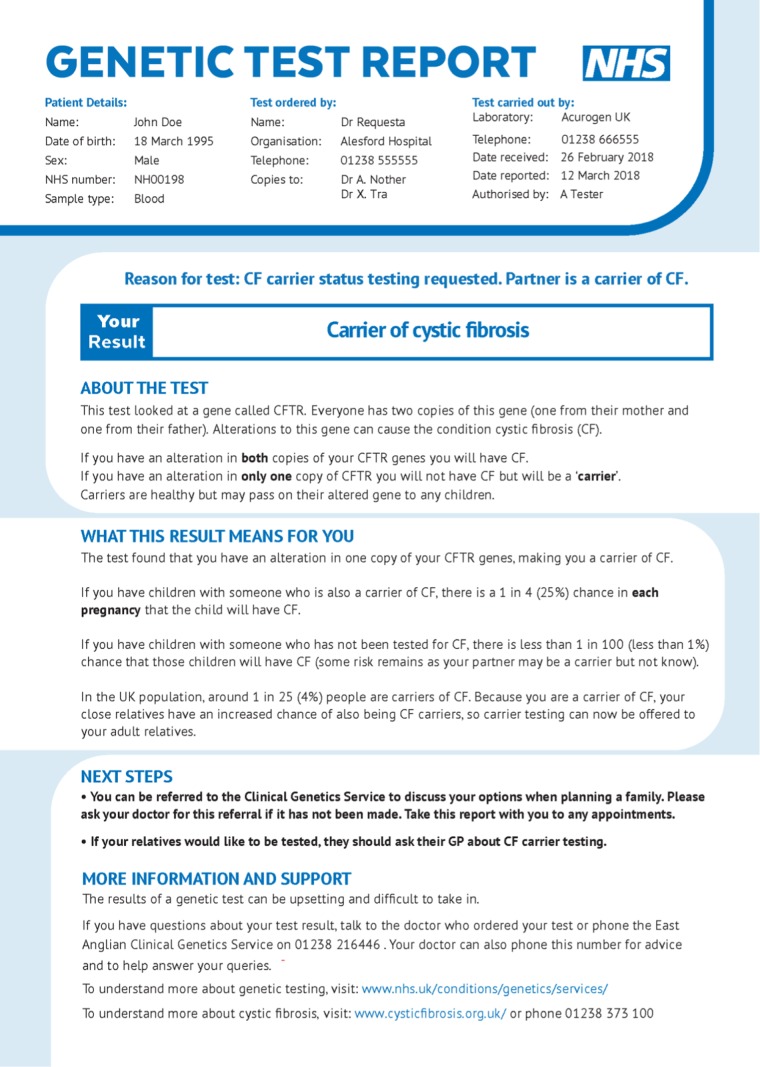
**Patient-friendly page of user-centered “Positive/Partner p.Phe508del” report.**

User-centered testing can take a formative or summative approach. Formative testing is conducted iteratively while a product is still in development, whereas summative testing is done once the stopping criterion has been met and the design finalized. Their goals differ accordingly: whereas “formative testing focuses on identifying ways of making improvements, summative testing focuses on evaluating against a set of criteria”.^33^ All five reports were subject to formative testing, and two were selected for summative testing, namely those having “partner heterozygous for p.Phe508del” as the reason for referral (Figs. S1, S2; sample patient-centered page in Fig. 1). Corresponding anonymized “standard” report templates currently in use were obtained from Yorkshire and North East Genomic Laboratory Hub Central Laboratory to act as a control comparison (Figs. S3, S4), with permission. Information that could have been used to identify the laboratory that the templates came from was fictionalized. Informed consent was obtained from all participants. This study received ethical approval from the Cambridge Psychology Research Ethics Committee (PRE.2018.077).

### User-centered design process

#### Interviews

Three rounds of semistructured interviews were conducted over Skype with a convenience sample of 30 volunteers recruited from the Cambridge Rare Disease Network, individuals who had participated in previous studies, and researcher contacts. Twelve, eight, and ten volunteers participated in each round, respectively. Volunteers were compensated with Amazon vouchers for £10. Interviews included questions pertaining to communication efficacy and subjective comprehension (e.g., questions about reports’ appearance, structure, confusing language, etc.), objective comprehension, and actionability. Demographic information for participants in each round is summarized in Table S1.

#### Formative evaluation

The primary goal of the formative evaluation was to identify and address the most serious usability problems with the reports, borrowing the definition of Lavery et al.:^34^ “an aspect of the system and/or a demand on the user which makes it unpleasant, inefficient, onerous or impossible for the user to achieve their goals in typical usage situations.” Given that typical goals when receiving a genetic report are to (1) understand the contents and (2) to take appropriate next steps if necessary (or to advise the patient of appropriate next steps), we treated as usability problems issues that caused confusion, left participants with incorrect impressions, generated unnecessary anxiety, or decreased the odds that a participant would be able to get the assistance they needed to take appropriate next steps. After rounds 2–3, interviewer notes and partial transcriptions of participants’ answers to interview questions were reviewed and coded in MaxQDA to identify and evaluate the most significant problems, highlight cases of poor comprehension, and assess the degree to which the reports met participants’ information needs. Full coding and partial transcription from interview recordings were completed post hoc for round 1, but interviewer notes were reviewed and usability problems were enumerated and corrected prior to round 2 nevertheless. Our stop criterion for how many rounds of interviewing to conduct was that by the final round, no major usability problems should remain. Major usability problems are those for which “the user will probably use or attempt to use the product, but will be severely limited in his or her ability to do so”;^35^ we considered these to include issues that could leave recipients with a serious misconception.

Because we ultimately wished to run a summative evaluation focusing on subjective comprehension, risk probability comprehension, and communication efficacy, we categorized participant answers to questions intended to highlight usability issues that might affect these constructs in particular, as well as more exploratory constructs of interest (e.g., actionability, the degree to which “consumers of diverse backgrounds and varying levels of health literacy can identify what they can do based on the information presented”^36^). These questions were asked to help determine whether there were problems in any of these domains so severe as to constitute a major usability problem.

#### Summative evaluation

Interviews were followed by an experiment in which participants were given either the new (2-page) user-centered report or a standard (1-page) report currently in clinical use (and representative of standard practice). Our approach was to provide participants with the entire user-centered report, but to ask questions specific to the first page of the report to ensure that the patient-facing page was the one being evaluated. After receiving the participant information sheet, a consent form, and background information about cystic fibrosis, study participants were presented with a clinical scenario in which a hypothetical John and Jane Doe are thinking about starting a family. Neither has cystic fibrosis, but CF runs in Jane’s family and she is known to be a carrier, so John’s general practitioner (GP) has advised him to have a carrier test to inform the couple’s family planning decisions. Participants were then shown a copy of “John’s report,” a report filled in with fictional information about Mr. Doe, and asked to read it carefully. The report shown was either one of the standard reports described earlier, or one of the new user-centered reports. The evaluation therefore had a 2 × 2 factorial between-participants design with two levels of design (standard and user-centered) and two levels of test result (positive and negative). Afterward, participants completed a questionnaire collecting outcome measures. On every questionnaire page, text stated “Please answer the following based on what you have learned from the first page of the report. To take another look at it, you may clickhere”; clicking brought up the first page of the report. Note that basic background information about cystic fibrosis was provided to bring the experimental scenario closer to a typical real-world scenario. This was not done within the reports themselves, as in the real world a couple with CF in one partner’s family would typically be aware of what CF is, particularly after meeting with a GP and being referred for testing.

#### Key outcomes were subjective comprehension, risk probability comprehension, and communication efficacy

Subjective comprehension was assessed by asking “How well did you understand the information in the first page of the report?” and “How clear is the information on the first page of the report?” on a seven-point scale ranging from 1 (“not at all”) to 7 (“completely”). Risk probability comprehension was assessed by tallying the number of risk probability comprehension questions answered correctly out of seven presented, counting responses within ±1% of the correct answer as “correct.” An investigator blinded to condition converted free-text responses to numbers. Communication efficacy was assessed using a version of the 18-item questionnaire developed by Scheuner et al.,^16^ modified so as to be appropriate for laypersons rather than clinicians (Table 1). A power analysis suggested 192 participants were required to achieve 80% power to detect an effect size *f* of 0.25 with intent to test main effects and two-way interactions via analysis of variance (ANOVA). Alpha was adjusted to 0.01, two-tailed, permitting us to look for differences in the three key outcomes described earlier at an α of 0.05 with adjustment for multiple hypothesis testing. Normality of residuals was assessed using the Shapiro–Wilk test (α = 0.05).

**Table 1:** Scores for the standard and user-centered reports^a^

	Standard report		User-centered report		
	Mean	SD	Mean	SD	*p* value
**Subjective comprehension, clarity, trust** (7-point scale)					
How well did you understand the information…	4.94	1.23	5.74	1.18	<0.001
How clear is the information…	4.65	1.31	5.78	1.20	<0.001
How much do you trust the information…	5.92	1.12	6.23	0.99	0.03
**Communication efficacy** (4-point scale modified from Scheuner et al.^16^)					
How satisfied are you with…					
The general format (look and feel)…	2.62	0.90	3.31	0.71	<0.001
The amount of information…	2.83	0.83	3.38	0.73	<0.001
The organization of the information…	2.68	0.88	3.29	0.71	<0.001
How easy is it to…					
Find the test result…	2.67	0.95	3.35	0.78	<0.001
Find information…to help with decision making?	2.42	0.90	3.10	0.78	<0.001
Understand the language used…	2.35	0.88	3.25	0.78	<0.001
Understand the test result presented…	2.53	0.89	3.34	0.76	<0.001
Understand the interpretation of the test result…	2.56	0.90	3.13	0.80	<0.001
How effectively does the first page of the report…					
Communicate the test result?	2.67	0.88	3.38	0.73	<0.001
Communicate what this test result means?	2.47	0.98	3.23	0.67	<0.001
Communicate the patient’s options (i.e., John’s options) having received this test result?	2.16	1.00	2.94	0.79	<0.001
Communicate the availability of information resources for the patient (i.e., John)?	1.98	0.96	2.95	0.86	<0.001
Communicate the availability of information resources for health professionals (i.e., John’s GP)?	2.10	0.92	2.87	0.82	<0.001
Inform medical decisions the patient (i.e., John) might have to make as a result of this test?	2.46	0.99	3.01	0.74	<0.001
Help you explain what the test result means to other people?	2.42	0.93	3.00	0.88	<0.001
Help you understand the medical issues relating to the result?	2.27	0.90	2.81	0.89	<0.001
Help you understand the genetic aspects of the result?	2.40	0.93	2.97	0.85	<0.001
Communicate any limitations of the test result?	1.83	0.83	2.69	0.96	<0.001
**Actionability** (7-point scale)					
How clear are you about the next steps that you could take…	4.40	1.60	5.53	1.35	<0.001
Do you feel you would have the necessary information to decide what to do next…	4.26	1.71	5.21	1.52	<0.001
How certain are you about what you would do next…	4.49	1.75	5.62	1.32	<0.001
Do you feel you would have the necessary professional support to decide what to do next…	4.45	1.53	5.47	1.30	<0.001
How ready would you feel to take any next steps…	4.27	1.72	5.21	1.38	<0.001

^a^To make the table more compact, ellipses (“…”) appearing in communication efficacy questions and subjective understanding/clarity/trust questions stand in for the phrase “in the first page of the report” (“of the first page of the report,” communication efficacy question 1). Ellipses appearing in actionability questions stand in for the phrase “if you had received this report in real life.”

ANOVA is fairly robust to violations of normality, but for severe violations nonparametric alternatives are sometimes applied. For example, the Mann–Whitney test compares the mean ranks of two samples, where the rank of a value is determined by ranking all values from low to high regardless of sample. Power analysis indicated that if this were used to compare the user-centered and standard reports on any of our key dependent variables, 192 participants would yield 78% power to detect a medium-sized effect (*d* = 0.5, α = 0.01). The Scheirer–Ray–Hare extension of the Kruskal–Wallis test^37^ is a nonparametric ANOVA alternative based on ranks rather than means; 192 participants would provide 78% power to detect medium-sized main effects (*f* = 0.25, α = 0.01).

Forty-eight participants were randomized by the Qualtrics survey distribution software to each combination of design (standard and user-centered) and test result (positive and negative), excepting positive user-centered, which had 49 due to a glitch with Prolific. “Difficult” risk probability comprehension questions always followed “easy” questions, but the order in which questions were presented was otherwise counterbalanced by question type (Table 2). Our minimum acceptable goal for the evaluation was to outperform the standard template on at least one key outcome without being inferior on the other two, although we hoped to outperform it significantly on all measures. Tests were two-sided with Bonferroni correction for multiple hypothesis testing. Measures of central tendency reported in “Results” are means, unless otherwise stated.

**Table 2:** Measures of participant comprehension of risk probabilities

Question ID/type and difficulty	Question	Answer format
Q1/Carrier risk (easy)	What do you think the probability is that John is a carrier of cystic fibrosis? You can indicate this probability as a percentage, or in another way if you prefer.	Free text
Q2/Carrier risk (easy)	Please indicate the probability that John is a carrier of cystic fibrosis by dragging the slider below.^a^	Probability slider from “0% chance” to “100% chance”
Q3/Risk to child (easy)	If John and Jane have a child, what do you think the probability is that the child will have cystic fibrosis? You can indicate this probability as a percentage, or in another way if you prefer.	Free text
Q4/Risk to child (easy)	Please indicate the probability that the child will have cystic fibrosis by dragging the slider below.^a^	Probability slider from “0% chance” to “100% chance”
Q5/Risk to child (hard)	Imagine that there are 1000 couples in exactly the same situation as John and Jane: that is to say, • one partner is a carrier (like Jane is), and • the other partner has had the same test that John has had, and received the same result as John did. If each of these 1000 couples have one child, about how many of these 1000 children would have cystic fibrosis? If you aren’t sure, or if you think there are many possibilities, please make your **best guess** as to the**most likely** number of children to have cystic fibrosis, from 0 to 1000.	Free text; single number expected
Q6/Risk to child (hard)	[As above with “800” in place of “1000”]	Free text; single number expected
Q7/Both risks	• Which of the following possibilities is more likely? • John Doe is a carrier of cystic fibrosis • The first child of John and Jane Doe will have cystic fibrosis	Multiple choice: • It’s more likely that John Doe is a carrier of cystic fibrosis • It’s more likely that the first child of John and Jane Doe will have cystic fibrosis • Both possibilities are equally likely • Don’t know

^a^The following text followed in both cases: “If you aren’t sure, please make your best guess. If you can’t mark exactly the probability you want using the slider, please put it as close to that probability as you can.”

A secondary goal was to achieve superiority on at least one measure (without being inferior on any measure), out of *all* measures recorded. This included not only key outcomes, but also five exploratory measures: trust, actionability, risk probability interpretation, visibility of result summary, and ease of understanding the result summary. Trust was assessed by asking “How much do you trust the information in the first page of the report?” on a 7-point scale from 1 (“not at all”) to 7 (“completely”), and five questions related to actionability were included (Table 1). Two risk probability interpretation questions were included—“Is John a carrier of cystic fibrosis?” and “If John and Jane have a child, will the child have cystic fibrosis?”—with multiple-choice answers (definitely not, unlikely, likely, and definitely). This provides insight into how people understand the numbers, but we had no goal beyond ensuring that viewers of positive reports did not conclude that the couple would “definitely” or “definitely not” have a child with CF, and that viewers of negative reports did not conclude that the couple would “likely” or “definitely” have a child with CF. This is because there is no right answer with respect to whether a 25% chance of having a child with cystic fibrosis feels “unlikely” or all too “likely.” Participants were asked whether they had noticed the result summary (the “Your Result” box for the user-centered report, or the analogous “Summary” statement for the standard report) and how easy the result was to understand (from 1 “not at all easy” to 7 “very easy”). Finally, subjective numeracy^38^ was collected, as well as demographic information.

## RESULTS

### Formative evaluation

Quantitative summaries of participant responses to questions relating to subjective comprehension, risk probability comprehension, communication efficacy, and actionability are provided in Figs. S5–S9 and Table S3. Answers to these questions suggested adequate comprehension of the version 3 reports, at least in our small sample of ten participants (Table S3). A summary of changes made after each round of testing is available in Tables S4 and S5, and qualitative description of usability problems in each round and severity classifications are given in Table S6, with nothing rising to the level of a major usability problem by the final round. Formative evaluation was therefore stopped at this point and a summative evaluation was conducted for the version 3 partner reports. A full analysis of all substantive participant comments is beyond the scope of this paper, but a few examples of how specific usability issues led to specific changes are detailed in Table S7.

One issue noted during round 3 was that multiple participants commented that they had not noticed the result summary box on their first read-through. This did not rise to the level of a usability problem as these participants all read and understood the description of the result in the “What This Result Means For You” section, but it was of sufficient concern that visibility of result summary was added to the summative evaluation as an exploratory measure.

### Summative evaluation

One hundred ninety-three participants were paid £1.96/person to complete the study via Prolific Academic; demographic characteristics appear in Table S2. Due to violations of normality, Mann–Whitney *U*-tests were used rather than ANOVAs, comparing mean ranks between the two conditions.

Subjective comprehension was higher for the user-centered (UC) reports, whether participants were asked about understanding (M_UC_ = 5.74, SD_UC_ = 1.18, M_standard_ = 4.94, SD_standard_ = 1.23, *U* = 2896, *p* < 0.001, *d* = 0.7) or clarity (M_UC_ = 5.78, SD_UC_ = 1.20, M_standard_ = 4.65, SD_standard_ = 1.31, *U* = 2322, *p* < 0.001, *d* = 0.9). No differences were observed in risk probability comprehension (M_UC_ = 4.95, SD_UC_ = 2.30, M_standard_ = 4.94, SD_standard_ = 2.31, *U* = 4618, *p* = 0.9, *d* = 0.0), and item-wise chi-squared tests revealed that no questions in Table 2 were answered correctly more frequently in one condition than the other. Like Scheuner et al.,^16^ we compared the mean total scores on communication efficacy, finding higher scores for the user-centered reports (M_UC_ = 3.11, SD_UC_ = 0.56, M_standard_ = 2.41, SD_standard_ = 0.7, *U* = 2045, *p* < 0.001, *d* = 1.1). Item-wise analyses found significant differences for each item in favor of the user-centered reports, all *p* < 0.001 (Table 1). Analogous *U*-tests comparing positive versus negative reports were conducted, none of which found significant results.

User-centered reports trended slightly higher with respect to trust (M_UC_ = 6.23, SD_UC_ = 0.99, M_standard_ = 5.92, SD_standard_ = 1.12, *U* = 3874, *p* = 0.03, *d* = 0.3), nonsignificant after correction for multiple hypothesis testing. They were reliably higher on actionability (M_UC_ = 5.41, SD_UC_ = 1.20, M_standard_ = 4.37, SD_standard_ = 1.47, *U* = 2733, *p* < 0.001, *d* = 0.8), with item-wise analyses favoring the new reports on every question (Table 1). Surprisingly, 27% reported that they had not noticed the result summary in the user-centered reports versus 8% in the standard reports, X^2^(1, *N* = 193) = 10.1, *p* = 0.001. However, estimates of John’s probability of being a carrier (Table 2, question 2) were no different, suggesting that this information was clear even to those who missed the summary (positive reports: median 100% both conditions, M_UC_ = 0.86, SD_UC_ = 0.29, M_standard_ = 0.80, SD_standard_ = 0.32, *U* = 1170, *p* > 0.9,*d* = 0.2; negative reports: median 1% both conditions, M_UC_ = 0.07, SD_UC_ = 0.16, M_standard_ = 0.07, SD_standard_ = 0.16, *U* = 1161, *p* > 0.9,*d* = 0.0). The user-centered reports’ result summaries were also rated easier to understand, M_UC_ = 6.05, SD_UC_ = 1.33, M_standard_ = 5.00, SD_standard_ = 1.66, *U* = 2876, *p* < 0.001, *d* = 0.7.

**Fig. 2:**
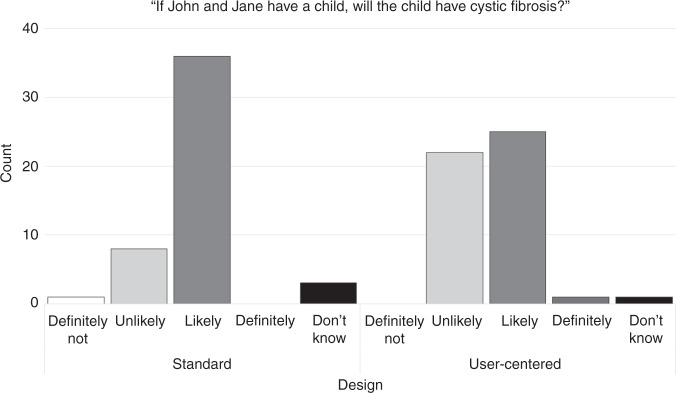
**Responses given by participants who viewed reports with positive test results to the question “If John and Jane have a child, will the child have cystic fibrosis?”** When asked to produce the numeric probability that the first child would have cystic fibrosis (Table 2, Section 4), participants who felt it was “likely” that the first child would have cystic fibrosis had mean estimates of 34% (SD = 21%) if they had seen the standard report, compared with 31% (SD = 12%) if they had seen the user-centered report (no significant difference, *U* = 473, *p* = 0.7). Participants who felt it was “unlikely” that the first child would have cystic fibrosis had mean estimates of 25% (SD = 0.4%) if they had seen the standard report, compared to 27% (SD = 14%) if they had seen the user-centered report (no significant difference, *U* = 100, *p* = 0.4).

When estimating the probability that the first child would have cystic fibrosis (Table 2, question 4), there were no significant differences between levels of design for either positive reports (median 25% both conditions; M_UC_ = 0.31, SD_UC_ = 0.16, M_standard_ = 0.33, SD_standard_ = 0.19, *U* = 1328, *p* = 0.2, *d* = −0.2) or negative reports (median 1% both conditions; M_UC_ = 0.10, SD_UC_ = 0.17, M_standard_ = 0.06, SD_standard_ = 0.11, *U* = 1100, *p* = 0.8, *d* = 0.3). Nevertheless, responses to the risk interpretation questions suggested possible differences in the interpretation of these numbers (Fig. 2) for those who had been shown the positive reports, with those who saw the user-centered positive report more apt to say that a child of two carriers was “unlikely” to have cystic fibrosis than those who saw the standard positive report, X^2^(1, *N* = 97) = 7.8, *p* = 0.005. Overall performance with respect to the goals of the evaluation is summarized in Table S8.

Despite the violations of normality, 2 × 2 ANOVAs crossing design with test result as well as the Scheirer–Ray–Hare extension of the Kruskal–Wallis test were also run on our key dependent measures. In both cases the same main effects were found, with no significant interactions.

## DISCUSSION

Our findings suggest that by starting with a patient-friendly generic report template and modifying it for a specific genetic test with a rapid user-centered design process, reports can be made that laypersons find significantly clearer, easier to understand, and more effective at communicating key information, including what they should do next (actionability). The improvements in actionability are particularly encouraging, as several interview participants noted that it is especially important that patients feel they understand “next steps,” and that they feel they have adequate information and support to make follow-up decisions. We also saw cautions from the risk comprehension literature^39^ borne out in our qualitative results (Table 3). Although we found no differences in risk probability comprehension, performance was near ceiling, with a median of 6 of 7 questions correct for both the user-centered and standard reports. Furthermore, combining user-centered testing with quantitative evaluation led us to insights that would have been difficult to achieve without both methods. For example, some individuals noted that although they understood their results from reading the text of the report, they had missed the summary box titled “Your Result.” Therefore, we added a question investigating this to our quantitative evaluation, which confirmed that 27% of participants did not remember seeing this box. Thus, even anecdotal evidence from small qualitative studies can generate important hypotheses that can then be tested more rigorously.

**Table 3:** Recommendations and lessons learned

Topic	Recommendation
Design	Splitting the design process into two phases—one to develop a generic template with key sections and information that patients want from the results, and one to populate that template with the specific numbers and information for each type of test—may provide an efficient way to produce large numbers of report templates for medical test results.
	Test with users: recommendations from the literature should not be applied blindly. For example, although there are good reasons to present risk figures in multiple formats as a general rule, in our case including “1 in 25 (4%)” and “1 in 4 (25%)” in close proximity caused confusion. User testing permitted us to address the issue in a way that allowed us to continue following the recommendation but also eliminated the confusion.
	Focus on recruitment of diverse representative end users throughout the process. We benefited from multiple perspectives of different user groups (health-care providers, patients, and members of the public with varying levels of experience of genetic testing), and would have benefited from a more concerted effort to recruit participants who were more diverse in other ways (e.g., education).
Evaluation	Following up on comments from interviews with a larger sample size can be a useful way to determine whether an offhand comment (“I don’t know how I missed that!”) is indicative of a larger issue (27% of participants indicating that they did not see the result summary box).
	Formative and summative evaluation both ought to be applied to important patient-facing materials whenever possible.
Vocabulary and wording	When using vocabulary that implies a change in risk (e.g., reduce/increase), the risks being compared must be clearly described.
	For patient-facing materials, “gene changes” is a poor plain-English alternative to “variant,” as it sometimes led to misinterpretations (e.g., “What does it mean by no cystic fibrosis gene changes detected? Can genes change throughout the life course or something? I thought you’re kind of born with it or you’re not.”) In our study, “alterations” seemed to be reasonably well received and interpreted.
	Prior literature^39^ has found that a quarter of people incorrectly answer the question “Which of the following numbers represents the biggest risk of getting a disease? 1 in 100, 1 in 1000, or 1 in 10?”, not realizing that a larger number in the denominator corresponds to a smaller probability. A quote from one of our participants suggested she had a similar misapprehension (“*less* than 1 in 500 sounds less scary, because then you can think, oh, it could be 400 or 200”). When presenting probabilities that are intended to be compared with each other, keep denominators constant to decrease the chances of misinterpretation, i.e., compare 1 in 1000 with 6 in 1000 rather than comparing 1 in 1000 with 1 in 167.

One limitation of our formative evaluation was that participants were overwhelmingly female (80%) and highly educated (Table S1). Our summative evaluation sample had similar biases (~69% female, ~56% university-educated), among other differences from the UK population (Table S2). Although subgroup analysis demonstrated that the benefits of our novel templates were thankfully not restricted to women, nor to the highly educated or highly numerate (Table S9), our development process could have identified important issues more quickly if we had solicited input from a more diverse group of participants from the outset. Given this nonrepresentative sample and the fact that it was more difficult to see the result summary in our report than in the standard report, we have made one additional change to address this, and are planning a replication of our summative evaluation with this new report using census-matched cross-stratified quota sampling.

Another drawback is that the use of a hypothetical scenario with our testing group means that our results are less likely to generalize than if they had been conducted as part of a clinical study. (See Stuckey et al.,^26^ Williams et al.^28^ for examples of patient-facing work that does not suffer from this limitation.) Furthermore, this study was limited to a single autosomal recessive condition. We have planned future research on reports for *BRCA1/BRCA2* testing, which will investigate whether the benefits of this approach generalize to material that is more challenging to communicate.

Overall, our experience demonstrated that a user-centered approach can be extremely helpful in discovering and rectifying usability problems with genetic reports. We hope that this research illustrates how rapid user-centered design can be used to develop more comprehensible and actionable reports, and that building on templates developed via user-centered design may be useful in developing patient-facing materials more generally.

## Supplementary information

Supplementary Figures legends

Supplementary Tables legends

## Data Availability

Code and data for primary analyses, as well as additional exploratory analyses not reported here, are available at https://github.com/WintonCentre/cf_reports (ver. 2019.07.22).
