# Correction: Creating genetic reports that are understood by nonspecialists: a case study

**DOI:** 10.1038/s41436-019-0663-2

**Published:** 2019-09-24

**Authors:** Gabriel Recchia, Antonia Chiappi, Gemma Chandratillake, Lucy Raymond, Alexandra L. J. Freeman

**Affiliations:** 1 Winton Centre for Risk and Evidence Communication, University of Cambridge, Cambridge, UK; 2 Institute of Continuing Education, University of Cambridge, Cambridge, UK; 3 East of England NHS Genomic Medicine Centre, London, UK; 4 Department of Medical Genetics, University of Cambridge, Cambridge, UK; 5 NIHR Bioresource-Rare Disease, London, UK

Correction to: *Genetics in Medicine* 2019; 10.1038/s41436-019-0649-0, published online 11 September 2019

This Article was originally published under Nature Research’s License to Publish, but has now been made available under a [CC BY 4.0] license. The PDF and HTML versions of the Article have been modified accordingly.

