# Supplementary material for: Creating genetic reports that are understood by nonspecialists: a case study
Source: Genet Med. 2019 Sep 11;22(2):353–61. doi: 10.1038/s41436-019-0649-0 (PMC7000324; doi:10.1038/s41436-019-0649-0)

Figure S1

User-centered "Positive / Partner p.Phe508del" report.

GENETIC TEST REPORT

NHS

Patient Details:

Name: John Doe
Date of birth: 18 March 1995
Sex: Male
NHS number: NH00198
Sample type: Blood

Test ordered by:

Name: Dr Requesta
Organisation: Alesford Hospital
Telephone: 01238 555555
Copies to: Dr A. Nother
Dr X. Tra

Test carried out by:

Laboratory: Acurogen UK
Telephone: 01238 666555
Date received: 26 February 2018
Date reported: 12 March 2018
Authorised by: A Tester

Reason for test: CF carrier status testing requested. Partner is a carrier of CF.

Your Result — Carrier of cystic fibrosis

ABOUT THE TEST

This test looked at a gene called CFTR. Everyone has two copies of this gene (one from their mother and one from their father). Alterations to this gene can cause the condition cystic fibrosis (CF).

If you have an alteration in **both** copies of your CFTR genes you will have CF.
If you have an alteration in **only one** copy of CFTR you will not have CF but will be a '**carrier**'.
Carriers are healthy but may pass on their altered gene to any children.

WHAT THIS RESULT MEANS FOR YOU

The test found that you have an alteration in one copy of your CFTR genes, making you a carrier of CF.

If you have children with someone who is also a carrier of CF, there is a 1 in 4 (25%) chance in **each pregnancy** that the child will have CF.

If you have children with someone who has not been tested for CF, there is less than 1 in 100 (less than 1%) chance that those children will have CF (some risk remains as your partner may be a carrier but not know).

In the UK population, around 1 in 25 (4%) people are carriers of CF. Because you are a carrier of CF, your close relatives have an increased chance of also being CF carriers, so carrier testing can now be offered to your adult relatives.

NEXT STEPS

- **You can be referred to the Clinical Genetics Service to discuss your options when planning a family. Please ask your doctor for this referral if it has not been made. Take this report with you to any appointments.**
- **If your relatives would like to be tested, they should ask their GP about CF carrier testing.**

MORE INFORMATION AND SUPPORT

The results of a genetic test can be upsetting and difficult to take in.

If you have questions about your test result, talk to the doctor who ordered your test or phone the East Anglian Clinical Genetics Service on 01238 216446 . Your doctor can also phone this number for advice and to help answer your queries.

To understand more about genetic testing, visit: www.nhs.uk/conditions/genetics/services/

To understand more about cystic fibrosis, visit: www.cysticfibrosis.org.uk/ or phone 01238 373 100

GENETIC TEST REPORT

NHS

Technical Information

Patient Details:

Name: John Doe
Date of birth: 18 March 1995
Sex: Male
NHS number: NH00198
Sample type: Blood
Sample collected: 10 March 2018
Sample received: 12 March 2018

For Your Records — **The information on this page is for health professionals. It is not essential that patients read this section.**

Gene	Zygosity	Inheritance	HGVS Description	Location	Classification
CFTR	Heterozygous	Autosomal recessive	NM_000492.3: c.1521_1523delCTT p.(Phe508del) GRCh37(hg19)	Chr7: g.117199646_117199648del	Pathogenic

Full Interpretation

John Doe's Partner, Jane (d.o.b. 28/09/2004) is a carrier of cystic fibrosis (CF) and is heterozygous for the common c.1521_1523delCTT p.(Phe508del) cystic fibrosis gene (CFTR) variant (information received with referral).

John Doe is heterozygous for the c.1521_1523delCTT p.(Phe508del) CFTR gene variant and is therefore a carrier of CF.

The risk of John Doe and Jane having a child with CF is 1 in 4 (25%) for each pregnancy. Prenatal diagnostic testing can be offered to this couple via referral to Clinical Genetics.

Carrier testing for the c.1521_1523delCTT p.(Phe508del) CFTR gene variant may be offered to John Doe's close relatives. For CF cascade testing in the East Anglian region, GPs can send a blood sample direct to the Regional Genetics Laboratory (www.cuh.nhs.uk/clinical-genetics). Relatives of the patient who live outside of East Anglia should ask their GP how testing is carried out locally.

Test Methodology

CF-EU2v1 Elucigene Assay (http://www.elucigene.com/product/cfeu2v1/) includes 50 of the most frequently observed European CF pathogenic variants - see our website (http://cuh.org.uk/genetics-labs-cf) for variant details. Variant nomenclature is in accordance with the Human Genome Variation Society (HGVS) guidelines according to GenBank accession number NM_000492.3 (DNA) and NP_000483.3 (protein) where +1 is the A of the ATG translation initiation codon.

Risk of a false positive: 0.1%

Risk of a false negative: 0.1%

References:

World Health Organization (WHO), 2004. The molecular epidemiology of cystic fibrosis. Available: http://www.who.int/genomics/publications/reports/en/

McCormick et al. 2002 Eur J Hum Genet. 2002 Oct;10(10):583-90.

Figure S2

User-centered “Negative / Partner p.Phe508del” report.

GENETIC TEST REPORT NHS

Patient Details:
Name: John Doe
Date of birth: 18 March 1995
Sex: Male
NHS number: NH00198
Sample type: Blood

Test ordered by:
Name: Dr Requesta
Organisation: Alesford Hospital
Telephone: 01238 555555
Copies to: Dr A. Nother, Dr X. Tra

Test carried out by:
Laboratory: Acurogen UK
Telephone: 01238 666555
Date received: 26 February 2018
Date reported: 12 March 2018
Authorised by: A Tester

Reason for test: CF carrier status testing requested. Partner is a carrier of CF.

Your Result	No common cystic fibrosis gene alterations detected

ABOUT THE TEST

This test looked at a gene called CFTR. Everyone has two copies of this gene (one from their mother and one from their father). Alterations to this gene can cause the condition cystic fibrosis (CF).

If you have an alteration in **both** copies of your CFTR genes you will have CF.
If you have an alteration in **only one** copy of CFTR you will not have CF but will be a ‘**carrier**’.
Carriers are healthy but may pass on their altered gene to any children.

WHAT THIS RESULT MEANS FOR YOU

Your sample has been tested for the common alterations to the CTFR gene that cause CF. No alterations were detected, so you are not a carrier of any of the alterations we tested for.

There are many alterations to the CFTR gene that are known to cause CF but some are very rare. This test looks for almost all of the alterations seen in the UK population.

The fact that the test did not detect any alterations means the chance that you carry a CFTR gene alteration is around 6 in 1000 (0.6%): there is still a very small possibility that you have one of the rare alterations. However, this is highly unlikely if there is no-one in your family who has CF.

If you have children with someone who is a carrier of CF, there is a less than 2 in 1000 (0.16%) chance in **each pregnancy** that the child will have CF.

NEXT STEPS

- **You do not need to do anything as a result of this test, but if you have questions about it, talk to your doctor.**

MORE INFORMATION AND SUPPORT

The results of a genetic test can be upsetting and difficult to take in.

If you have questions about your test result, talk to the doctor who ordered your test or phone the East Anglian Clinical Genetics Service on 01238 216446. Your doctor can also phone this number for advice and to help answer your queries.

To understand more about genetic testing, visit: www.nhs.uk/conditions/genetics/services/

To understand more about cystic fibrosis, visit: www.cysticfibrosis.org.uk/ or phone 01238 373 100

GENETIC TEST REPORT NHS

Technical Information

Patient Details:
Name: John Doe
Date of birth: 18 March 1995
Sex: Male
NHS number: NH00198
Sample type: Blood

Sample collected: 10 March 2018
Sample received: 12 March 2018

For Your Records — **The information on this page is for health professionals. It is not essential that patients read this section.**

Gene	Zygosity	Inheritance	HGVS Description	Location	Classification
CFTR	No variant	Not applicable	NM_000492.3: c.1521_1523delCTT p.(Phe508del) GRCh37(hg19)	Not applicable	Not applicable

Full Interpretation

John Doe’s Partner, Jane (d.o.b. 28/09/2004) is a carrier of cystic fibrosis (CF) and is heterozygous for the common c.1521_1523delCTT p.(Phe508del) cystic fibrosis gene (CFTR) variant (information received with referral).

DNA from John Doe has been tested for the common c.1521_1523delCTT p.(Phe508del) CFTR gene variant as well as other CFTR variants (see below) which together account for approximately 85% of UK CFTR gene variants (WHO, 2004). No CFTR gene variants were detected by our test.

This reduces John Doe’s CF carrier risk to 1 in 161 (0.6%).

Test Methodology

CF-EU2v1 Elucigene Assay (http://www.elucigene.com/product/cfeu2v1/) includes 50 of the most frequently observed European CF pathogenic variants - see our website (http://cuh.org.uk/genetics-labs-cf) for variant details. Variant nomenclature is in accordance with the Human Genome Variation Society (HGVS) guidelines according to GenBank accession number NM_000492.3 (DNA) and NP_000483.3 (protein) where +1 is the A of the ATG translation initiation codon.

Risk of a false positive: 0.1%

Risk of a false negative: 0.1%

References:

World Health Organization (WHO), 2004. The molecular epidemiology of cystic fibrosis. Available: http://www.who.int/genomics/publications/reports/en/

McCormick et al. 2002 Eur J Hum Genet. 2002 Oct;10(10):583-90.

Figure S3

Standard “Positive / Partner p.Phe508del” report.

Acurogen Genetics Laboratory

101 Long Street, Alesford, Devon, AS1 1AB, UK

Website: www.acurogen.co.uk
Email: info@acurogen.co.uk

Clinical Service Lead:
Dr H Ead PhD FRCPath

Tel: 01238 555666
Fax: 01238 666555

Report of Molecular Genetic Analysis for Cystic Fibrosis

Patient name: **DOE** John

DOB: 18 Mar 1995

Sex: Male

NHS No.: NH00198

Unit no.: Not provided

Sample type: Blood

Referred by: Dr Requesta

Unit:

Date requested: 26 Feb 2018

Acurogen Pedigree:

Acurogen Lab no:

Test reason: Partner is a CF carrier (information provided with referral; no further information available). This patient is assumed to be at ~1 in 25 prior risk of being a CF carrier (population carrier risk). The prior risk to any future child of this patient and their partner of being affected with CF is ~1 in 100.
Carrier test.

Results and Interpretation:

Test	Result	Lab Ref
CF-EU2v1	**c.1521_1523del heterozygote**	ACU55-5555

CF-EU2v1 analysis indicates that this patient is heterozygous for the pathogenic CFTR mutation c.1521_1523del p.(Phe508del). The remaining CFTR mutations tested for by the CF-EU2v1 kit are absent. This patient is therefore a CF carrier. The risk to any future child of this patient and their partner of being affected with CF is increased to 1 in 4. Assuming that this patient's partner has an identified CFTR mutation, prenatal CF testing is available if appropriate. We strongly recommend that this patient and their partner are referred to their local Clinical Genetics department.

This result also has important implications for relatives of this patient, and testing is available if appropriate. We recommend that these individuals are referred to their local Clinical Genetics department.

Summary:

This patient is heterozygous for the pathogenic CFTR mutation c.1521_1523del, and is therefore a CF carrier. The risk to any future child of this patient and their partner of being affected with CF is increased to 1 in 4.

Reported: Dr A Filer — *Clinical Scientist*

Authorised: Dr A Tester — *Principal Clinical Scientist*

Date: 12 Mar 2018

All reports depend upon the diagnosis of affected individuals, identification of samples and biological relationships of the individuals being correct.

Notes: Germline mutations within the CFTR gene cause cystic fibrosis (CF) or a CFTR-related disorder (CFTR-RD), which have autosomal recessive inheritance. The CF-EU2v1 (Elucigene) kit uses fluorescent ARMS (Amplification Refractory Mutation System) allele-specific amplification technology to identify 50 CFTR point mutations, insertions, or deletions. Please see the laboratory website (http://www.acurogen.co.uk) for a list of these 50 mutations, including previous nomenclature. In the local Caucasian population these mutations account for approximately 88% of all CF alleles. CFTR mutations are named according to HGVS (www.hgvs.org) guidelines using the reference sequence NM_000492.3. Very rare variants within the CFTR gene may interfere with the CF-EU2v1 assay, causing false positive or false negative results.
Please see the laboratory website (http://www.acurogen.co.uk) for further information regarding CFTR analysis.

References:
Schwarz et al. Cystic fibrosis mutation analysis: Report from 22 UK regional genetics laboratories. Hum Mutat. 1995;6(4):326-33
McCormick et al. Demographics of the UK cystic fibrosis population: Implications for neonatal screening. Eur J Hum Genet. 2002 Oct;10(10):583-90

NHS Devon Partnership NHS Trust

Figure S4
Standard "Negative / Partner p.Phe508del" report.

Acurogen Genetics Laboratory

101 Long Street, Alesford, Devon, AS1 1AB, UK

Website: www.acurogen.co.uk
Email: info@acurogen.co.uk

Clinical Service Lead:
Dr H Ead PhD FRCPath

Tel: 01238 555666
Fax: 01238 666555

Report of Molecular Genetic Analysis for Cystic Fibrosis

Patient name: **DOE** John
DOB: 18 Mar 1995
Sex: Male
NHS No.: NH00198
Unit no.: Not provided
Sample type: Blood

Referred by: Dr Requesta
Unit:
Date requested: 26 Feb 2018

Acurogen Pedigree:
Acurogen Lab no:

Test reason: Partner is a CF carrier (information provided with referral; no further information available). This patient is assumed to be at ~1 in 25 prior risk of being a CF carrier (population carrier risk). The prior risk to any future child of this patient and their partner of being affected with CF is ~1 in 100.
Carrier test.

Results and Interpretation:

Test	Result	Lab Ref
CF-EU2v1	**c.1521_1523del heterozygote**	ACU55-5555

Analysis indicates that the 50 CFTR mutations tested for by the CF-EU2v1 kit are absent in this patient. This result reduces this patient's CF carrier risk to ~1 in 200. The risk to the first child of this patient and their partner, Jane Doe, of being affected with CF is therefore reduced to ~1 in 800. Prenatal testing is not indicated.

Summary:

This patient's CF carrier risk is reduced to ~1 in 200. The risk to the first child of this couple of being affected with CF is reduced to ~1 in 800.

Reported: Dr A Filer
Clinical Scientist

Authorised: Dr A Tester
Principal Clinical Scientist

Date: 12 Mar 2018

All reports depend upon the diagnosis of affected individuals, identification of samples and biological relationships of the individuals being correct.

Notes: Germline mutations within the CFTR gene cause cystic fibrosis (CF) or a CFTR-related disorder (CFTR-RD), which have autosomal recessive inheritance. The CF-EU2v1 (Elucigene) kit uses fluorescent ARMS (Amplification Refractory Mutation System) allele-specific amplification technology to identify 50 CFTR point mutations, insertions, or deletions. Please see the laboratory website (http://www.acurogen.co.uk) for a list of these 50 mutations, including previous nomenclature. In the local Caucasian population these mutations account for approximately 88% of all CF alleles.
CFTR mutations are named according to HGVS (www.hgvs.org) guidelines using the reference sequence NM_000492.3. Very rare variants within the CFTR gene may interfere with the CF-EU2v1 assay, causing false positive or false negative results.
Please see the laboratory website (http://www.acurogen.co.uk) for further information regarding CFTR analysis.

References:
Schwarz et al. Cystic fibrosis mutation analysis: Report from 22 UK regional genetics laboratories. Hum Mutat. 1995;6(4):326-33
McCormick et al. Demographics of the UK cystic fibrosis population: Implications for neonatal screening. Eur J Hum Genet. 2002 Oct;10(10):583-90

Figure S5

Participant answers to the question "Is the patient given enough guidance about the implications of the result and what actions they could now take?" were coded by 3 independent raters (Fleiss' kappa 0.6) into the categories indicated in the legend. Visualisation on left panel includes all participants, while right panel excludes healthcare providers.

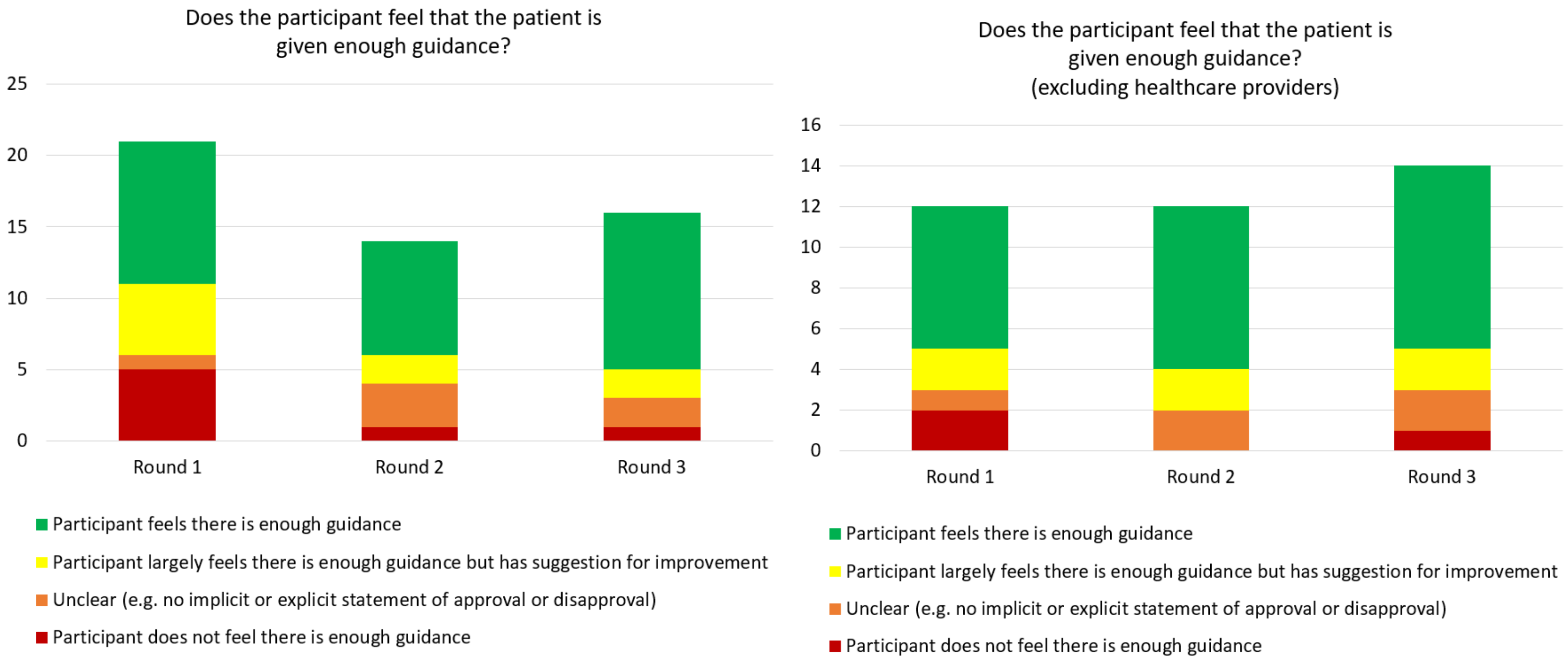

Figure S6

Participant answers to the question "What are your thoughts on the wording of the report and the level of the language used on page 1?" were coded for *participant approval* by 3 independent raters (Fleiss' kappa 0.7) into the categories indicated in the legend. Visualisation on left panel includes all participants, while right panel excludes healthcare providers.

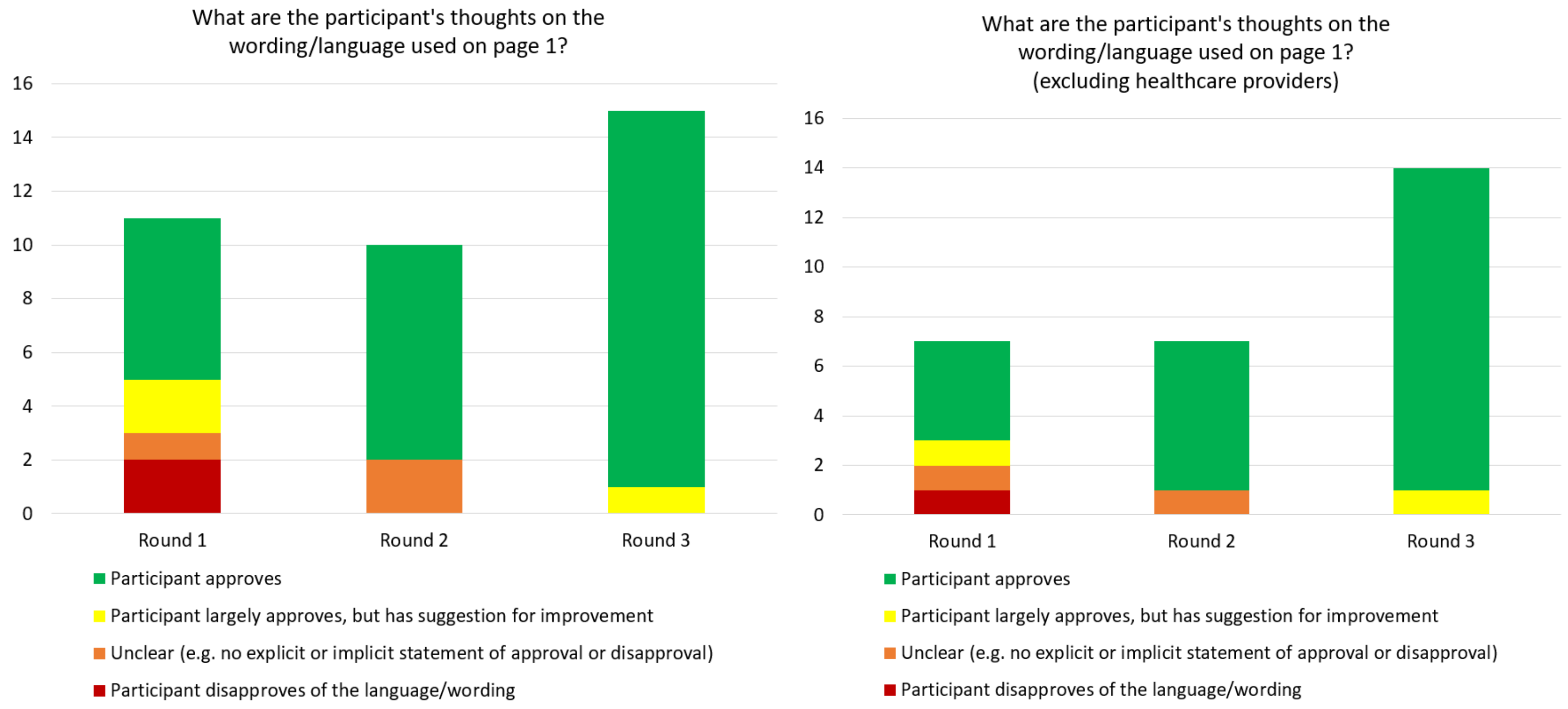

Figure S7

Participant answers to the question “What are your thoughts on the wording of the report and the level of the language used on page 1?” were coded for *confusion* by 3 independent raters (Fleiss’ kappa 0.6) into the categories indicated in the legend. Visualisation on left panel includes all participants, while right panel excludes healthcare providers.

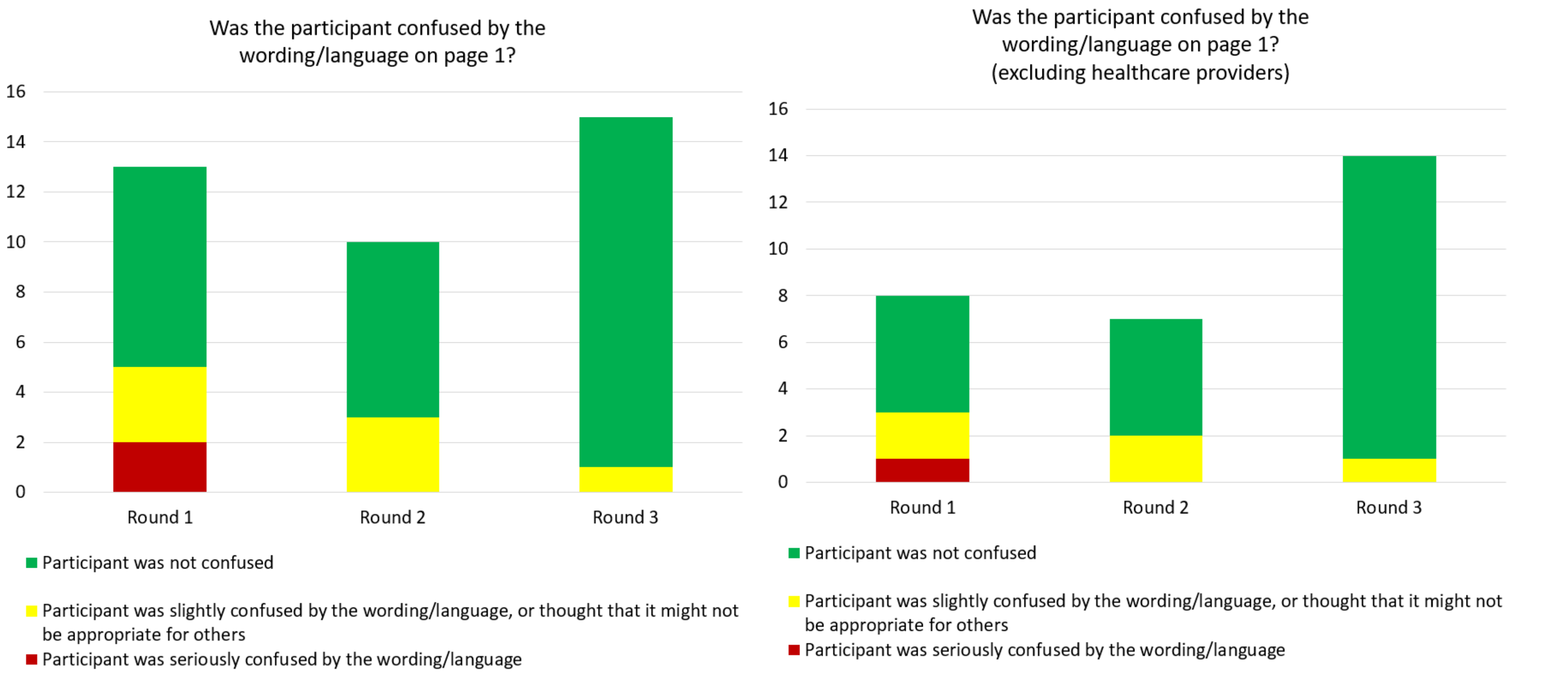

Figure S8

Participant answers to the question "Are there any particular words or phrases that are confusing?" were coded by 3 independent raters (Fleiss' kappa 0.8) into the categories indicated in the legend. Visualisation on left panel includes all participants, while right panel excludes healthcare providers.

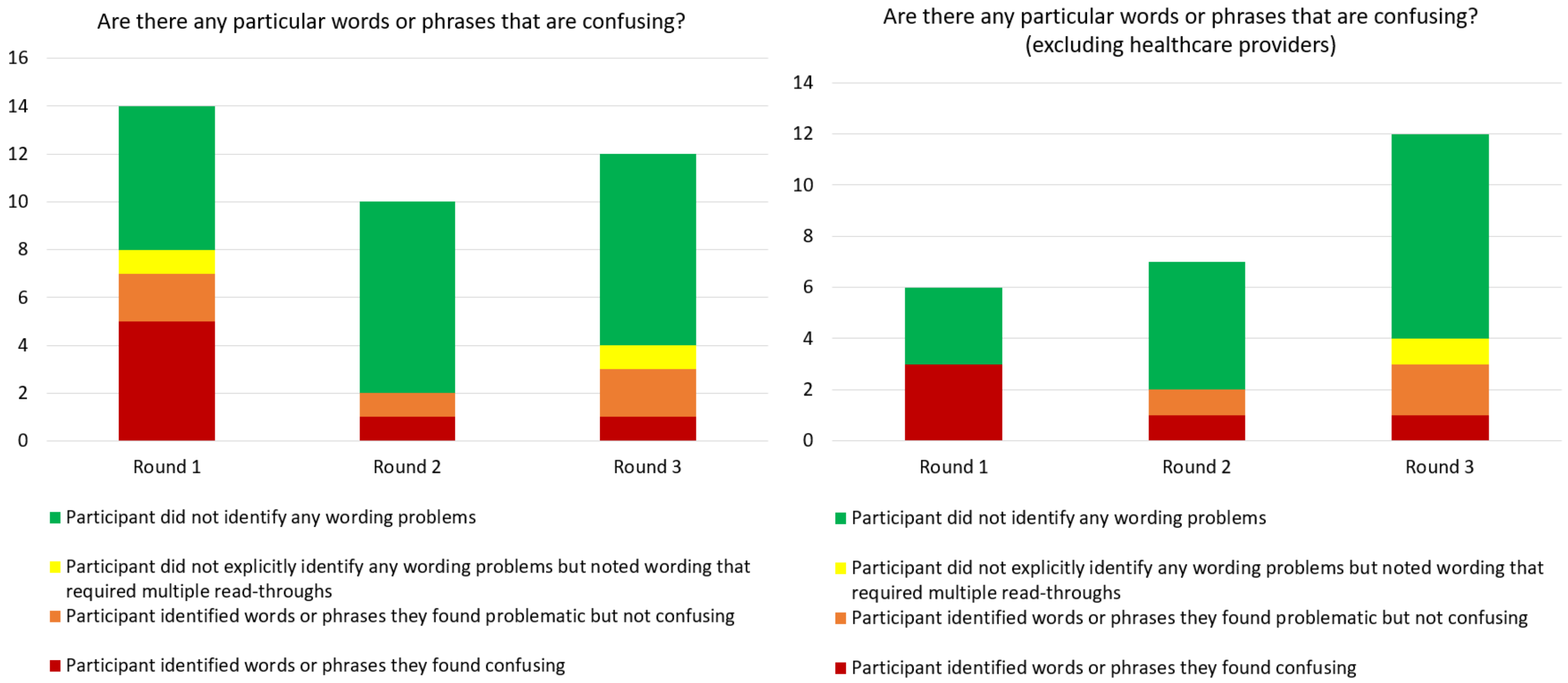

Figure S9

Participant answers to the question “Is there any information that you’d be left wanting to know after reading this report?” were coded by 3 independent raters (Fleiss’ kappa 0.9) into the categories indicated in the legend. Visualisation on left panel includes all participants, while right panel excludes healthcare providers.

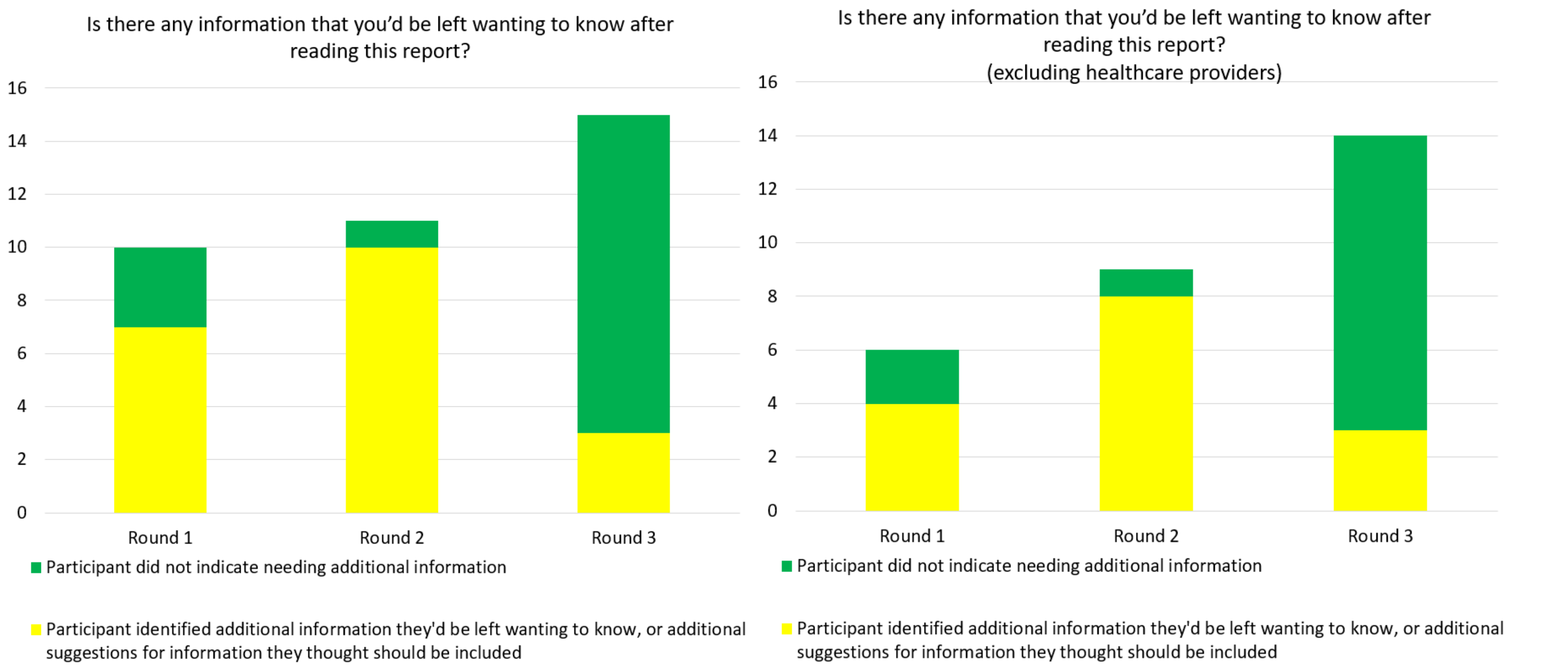

Figure S10

Density plot of communication efficacy.

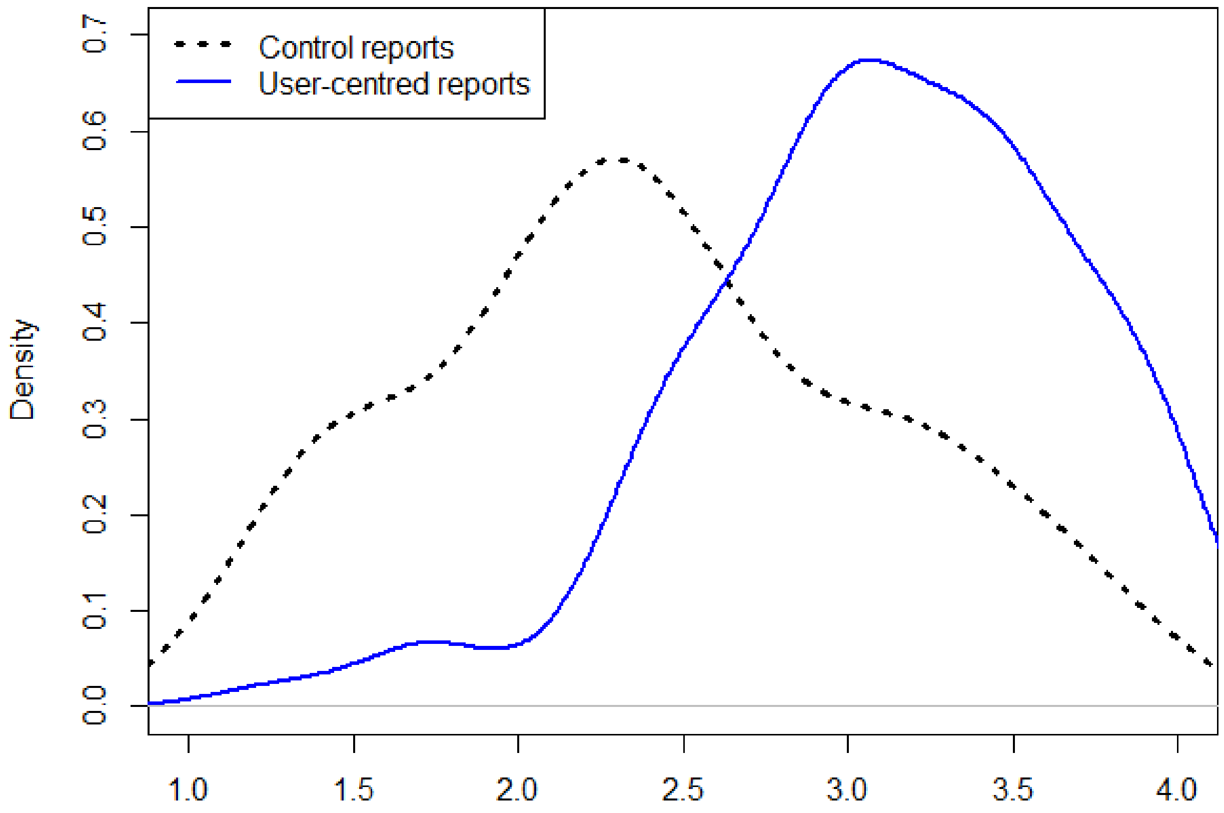

Figure S11

Density plot of subjective comprehension.

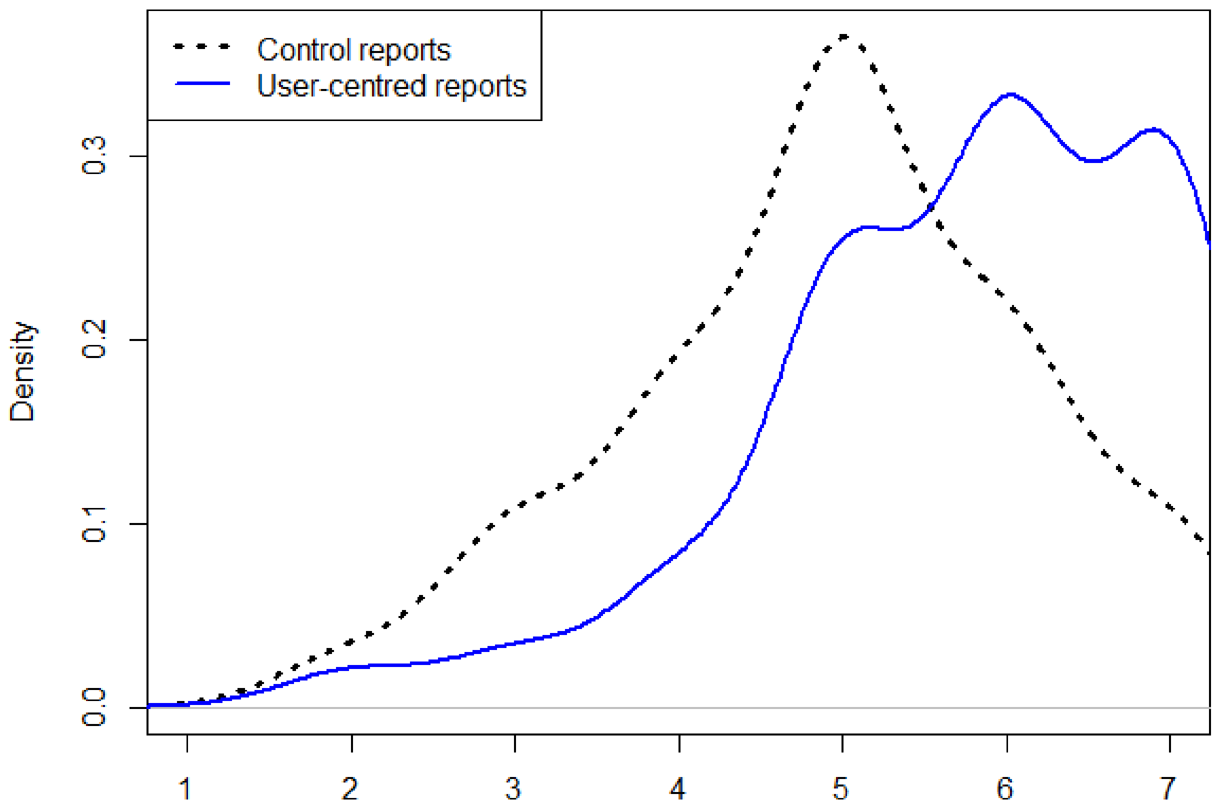

Figure S12

Density plot of objective comprehension.

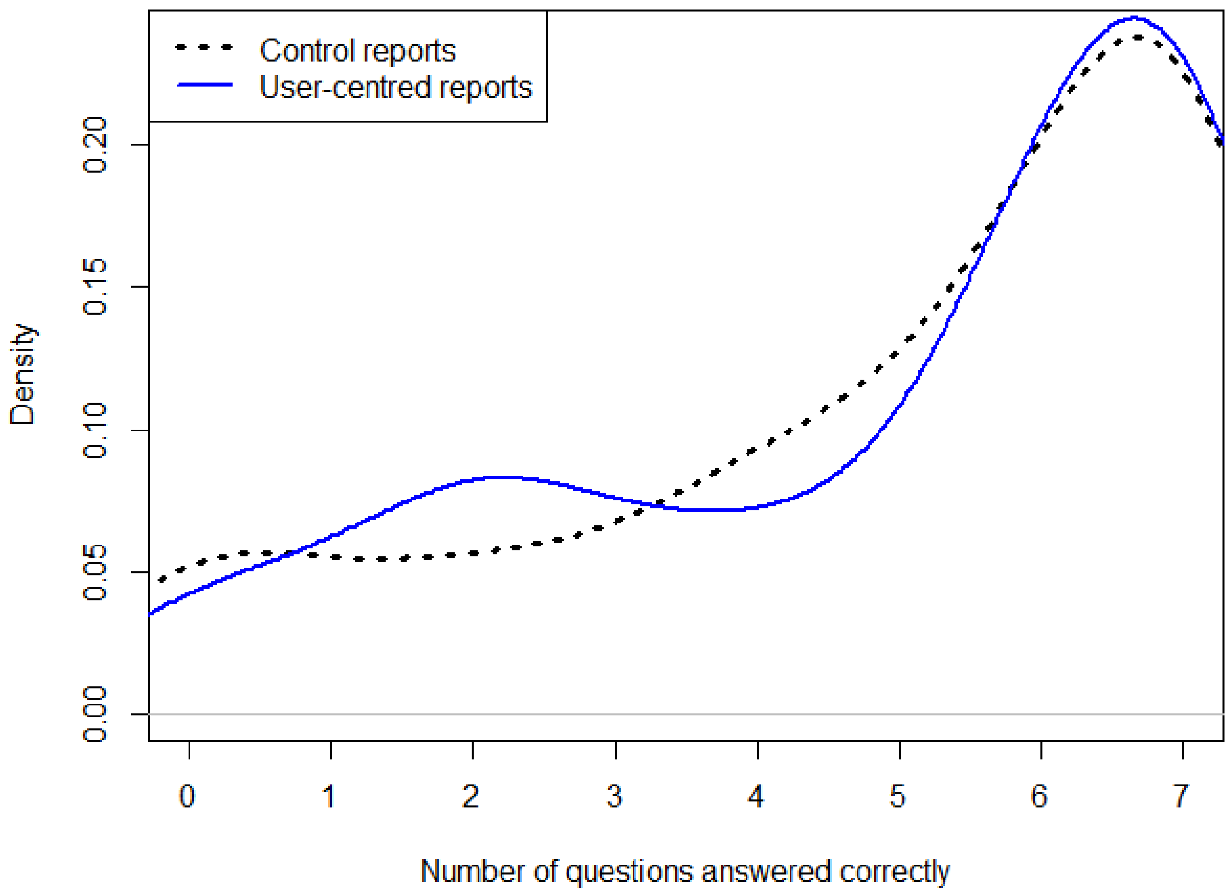

Figure S13

Density plot of subjective clarity.

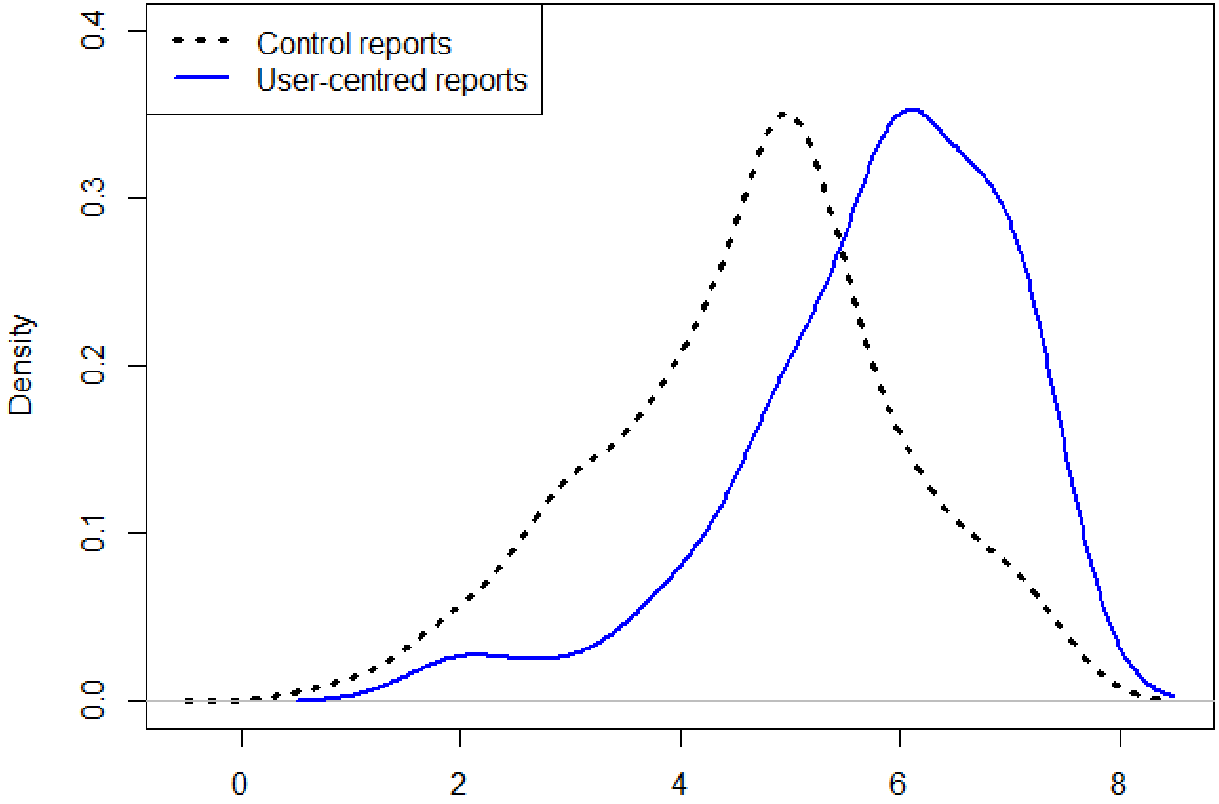

Figure S14

Density plot of subjective trust.

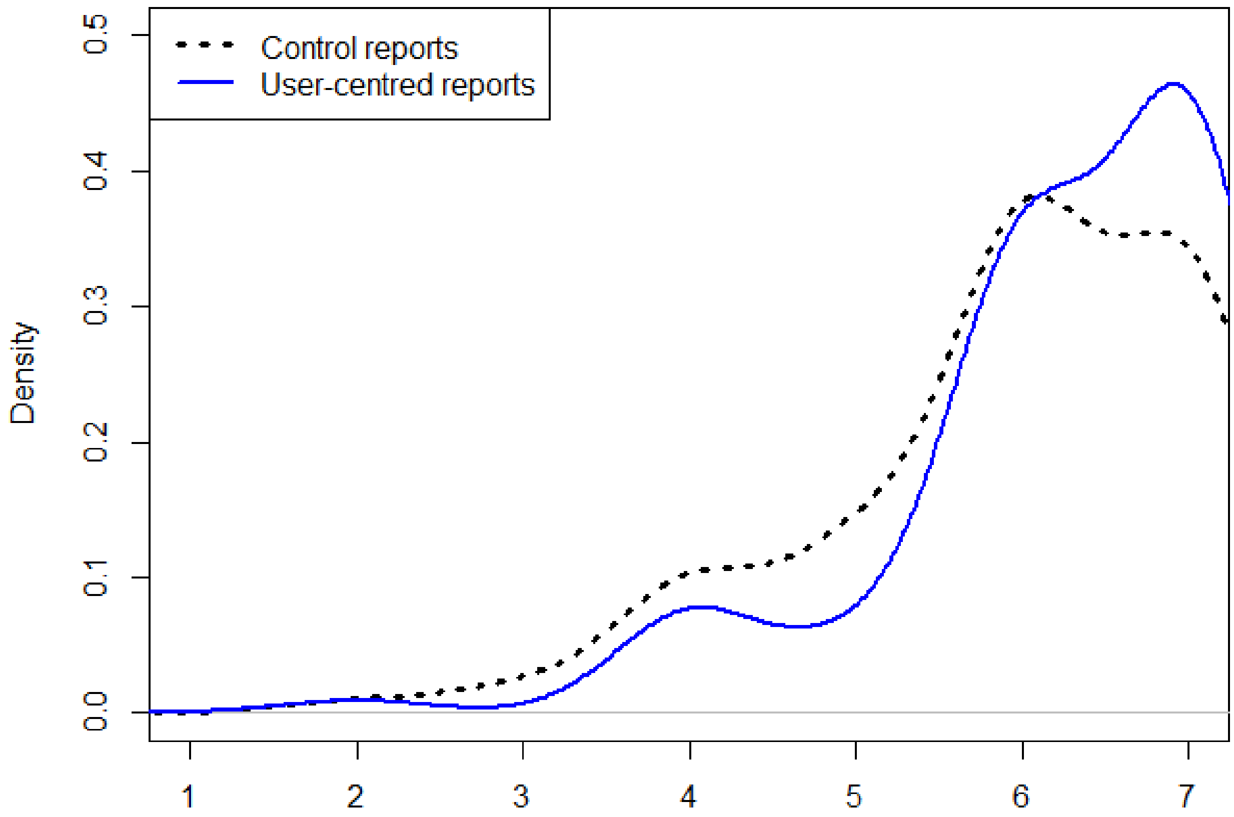

Figure S15

Density plot of actionability.

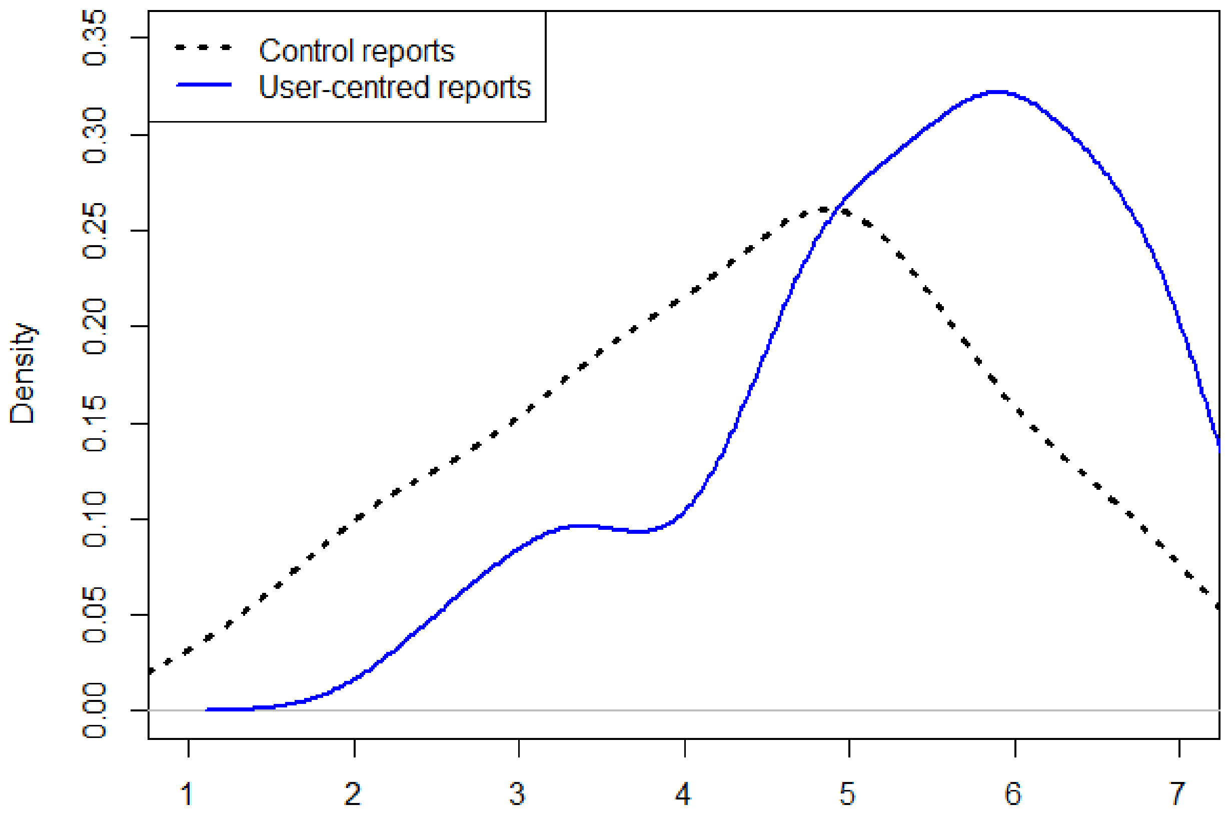

Figure S16

Density plot of ease of understanding of the result summary.

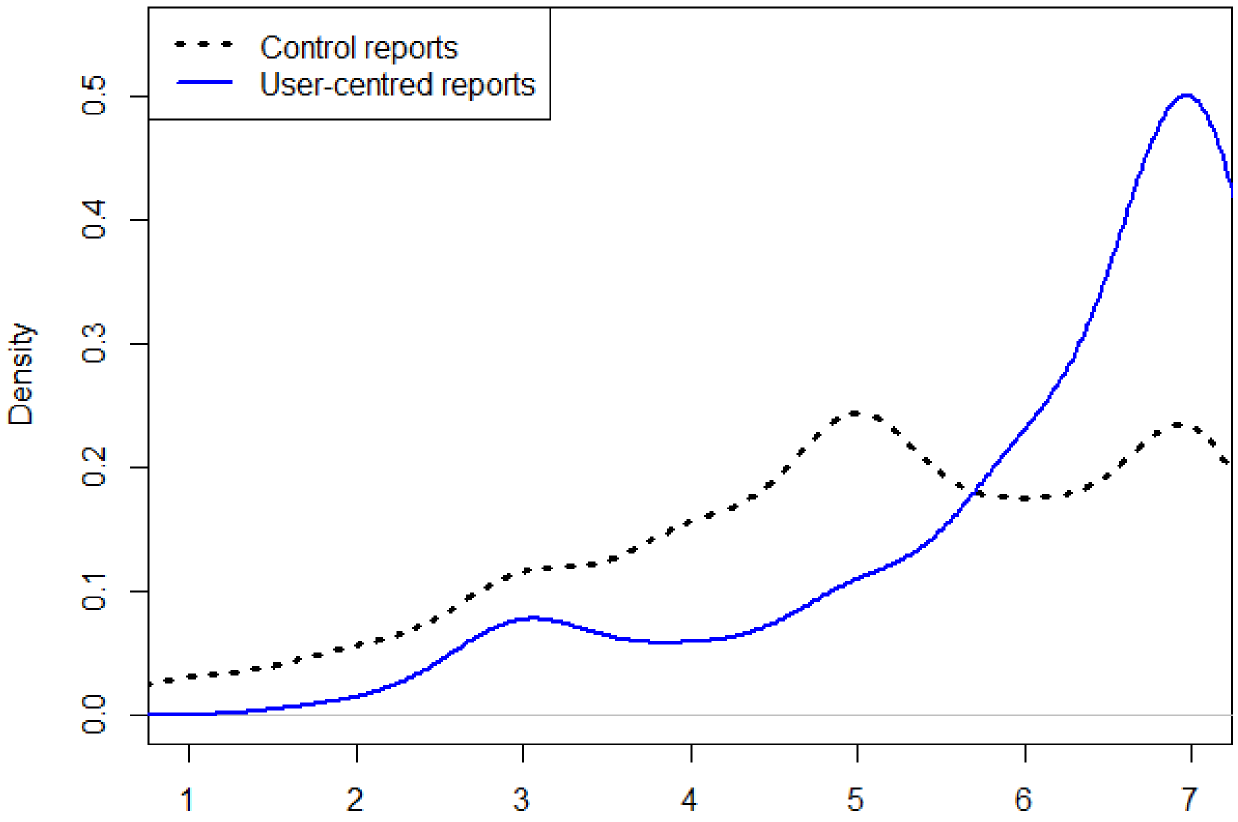

Supplement: Supplementary file 1 — Supplementary Figures legends [file 41436_2019_649_MOESM1_ESM.pdf]
