# Supplementary material for: Creating genetic reports that are understood by nonspecialists: a case study
Source: Genet Med. 2019 Sep 11;22(2):353–61. doi: 10.1038/s41436-019-0649-0 (PMC7000324; doi:10.1038/s41436-019-0649-0)
Supplement: Supplementary file 2 — Supplementary Tables legends [file 41436_2019_649_MOESM2_ESM.docx]

Table S1. Demographics of participants in formative evaluation.

|  | Round 1 | | Round 2 | | Round 3 | |
| --- | --- | --- | --- | --- | --- | --- |
| Participants | 12 | | 8 | | 10 | |
| Age range | 22-51 | | 26-46 | | 19-52 | |
| Gender |  | |  | |  | |
| Male | 4 | (33%) | 1 | (12%) | 1 | (10%) |
| Female | 8 | (67%) | 7 | (88%) | 9 | (90%) |
| Education |  | |  | |  | |
| No university | 2 | (17%) | 1 | (12%) | 3 | (30%) |
| Bachelors | 4 | (33%) | 3 | (38%) | 6 | (50%) |
| Post-graduate | 6 | (50%) | 4 | (50%) | 1 | (10%) |
| Health professional? |  | |  | |  | |
| Yes | 5 | (42%) | 2 | (25%) | 1 | (10%) |
| No | 7 | (58%) | 6 | (75%) | 9 | (90%) |
| Indicated that they themselves had CF or relative with CF? *^a^* |  |  |  |  |  |  |
| No | 12 | (100%) | 8 | (100%) | 10 | (100%) |

*^a^*Participants were not explicitly asked whether they had CF or had a relative with CF, but were asked whether they had experience with cystic fibrosis. 3 participants in Round 1 had cared for patients with CF, 1 indicated having known someone with CF, and 1 mentioned learning about it at school. 1 participant in Round 2 mentioned encountering CF in a clinical science rotation. 1 participant in Round 3 mentioned encountering CF as part of medical studies, and 1 participant mentioned knowing individuals with CF.

Table S2. Demographics of participants in summative evaluation.

|  | N | % | % in UK population, where known |
| --- | --- | --- | --- |
| Gender |  |  |  |
| Male | 55 | (28%) | 49% *^a^* |
| Female | 134 | (69%) | 51% *^a^* |
| Missing | 4 | (2%) |  |
| Age group |  |  |  |
| 18-24 years | 54 | (28%) | 31% *^b^* |
| 25-34 years | 62 | (32%) | 13% *^b^* |
| 35-44 years | 38 | (20%) | 14% *^b^* |
| 45-54 years | 27 | (14%) | 14% *^b^* |
| 55+ | 8 | (4%) | 28% *^b^* |
| Missing | 4 | (2%) |  |
| Adults in house |  |  |  |
| 1 | 30 | (16%) | 20% *^c^* |
| 2 | 106 | (55%) | 53% *^c^* |
| 3 | 30 | (16%) | 16% *^c^* |
| 4+ | 27 | (14%) | 11% *^c^* |
| Children in house |  |  |  |
| 0 | 30 | (16%) | As of 2017, 57% of people in UK households lived in households with one or more children *^d^* |
| 1 | 106 | (55%) |  |
| 2 | 30 | (16%) |  |
| 3 | 17 | (9%) |  |
| 4+ | 10 | (5%) |  |
| Combined income |  |  |  |
| Less than £10k | 20 | (10%) | As of 2017/18 financial year, quintiles of disposable UK household income were £13k, £22k, £29k, £38k, and £69k*^e^* |
| £10k to £20k | 28 | (14%) |  |
| £21k to £30k | 35 | (18%) |  |
| £31k to £40k | 26 | (14%) |  |
| £41k to £50k | 18 | (9%) |  |
| £51k to £60k | 18 | (9%) |  |
| £61k to £70k | 15 | (8%) |  |
| £71k to £80k | 5 | (3%) |  |
| £81k to £90k | 7 | (4%) |  |
| More than £91k | 11 | (6%) |  |
| Missing | 10 | (5%) |  |
| Education |  |  |  |
| GCSE or equivalent (e.g., level 2 NVQ) | 22 | (11%) | Primary/middle school, 19%; GCSE or A-levels, 35%; bachelors and higher, 46% *^f^* |
| A-Level or equivalent (e.g., IB or level 3 NVQ) | 61 | (32%) |  |
| Bachelors (e.g., BA, Bsc) | 81 | (42%) |  |
| Masters (e.g., MA, MSc) | 21 | (11%) |  |
| Doctoral (e.g., PhD) | 5 | (3%) |  |
| Missing | 3 | (2%) |  |
| Subjective numeracy (1 to 6) |  |  |  |
| Less than or equal to 3 | 21 | (11%) | Unknown in UK population. In one large study stratified to mirror the U.S. population, 25^th^ percentile was 3.2, median was 4.2, and 75^th^ percentile was 4.8*^g^* |
| 3.01 to 4 | 52 | (27%) |  |
| 4.01 to 5 | 70 | (36%) |  |
| 5.01 or greater | 50 | (26%) |  |
| Do you have any personal experience with cystic fibrosis? |  |  |  |
| Yes | 8 | (4%) |  |
| No | 185 | (96%) |  |

*^a^* *Male and female populations: GOV.UK ethnicity facts and figures*, 2018. Available at: https://www.ethnicity-facts-figures.service.gov.uk/uk-population-by-ethnicity/demographics/male-and-female-populations/latest. Accessed June 18, 2019.

*^b^* Percentages calculated from *Age groups: GOV.UK ethnicity facts and figures*, 2019. <https://www.ethnicity-facts-figures.service.gov.uk/uk-population-by-ethnicity/demographics/age-groups/latest>. Accessed June 18, 2019.

*^c^* Percentage of adults living in *N-*adult households calculated from *CT0774_2011 Census - Age of Household Reference Person (HRP) by number of adults in household - national to local authority leve*l. London: Office for National Statistics, 2018. Calculation excludes the 165 households in England and Wales with 15+ adults (less than .01% of all households). <https://www.ons.gov.uk/peoplepopulationandcommunity/housing/adhocs/008208ct07742011censusageofhouseholdreferencepersonhrpbynumberofadultsinhouseholdnationaltolocalauthoritylevel>. Accessed June 18, 2019.

*^d^* Calculated from *Families and households.* London: Office for National Statistics, 2017. <https://www.ons.gov.uk/peoplepopulationandcommunity/birthsdeathsandmarriages/families/datasets/familiesandhouseholdsfamiliesandhouseholds>. Accessed June 18, 2019.

*^e^* From Table 2 of *The effects of taxes and benefits on household income, disposable income estimate: 2018.* London: Office for National Statistics, 2019. <https://www.ons.gov.uk/peoplepopulationandcommunity/personalandhouseholdfinances/incomeandwealth/bulletins/householddisposableincomeandinequality/yearending2018/relateddata>

*^f^* OECD. Table A1.1 - Educational attainment of 25-64 year-olds (2017): Percentage of adults with a given level of education as the highest level attained", in *The Output of Educational Institutions and the Impact of Learning*, OECD Publishing, Paris, 2018: 54. <https://doi.org/10.1787/eag-2018-table14-en>. Following up on methodology referenced in table notes, and combining this with UK-specific definitions of ISCED levels (<http://gpseducation.oecd.org/Content/MapOfEducationSystem/GBR/GBR_2011_EN.pdf>) reveals that attainment of GCSE or A-levels corresponds to columns 5 and 6.

*^g^* Zikmund-Fisher BJ, Smith DM, Ubel PA, Fagerlin A. Validation of the subjective numeracy scale: Effects of low numeracy on comprehension of risk communications and utility elicitations. *Medical Decision Making* 2007 Sep; 27(5):663-71.

Table S3. Risk comprehension questions presented in rounds 2 and 3 of interviews.

| Question | Correct answer | Number answering correctly, round 2 | Number answering correctly, round 3 |
| --- | --- | --- | --- |
| For a random person in the UK who has never been tested for CF, what are their chances of being a carrier? | 1 in 25 (4%) | 7 of 8 (first answer given)  8 of 8 (after self-correction) | 10 of 10 (first answer given)  10 of 10 (after self-correction) |
| And what are their chances of not being a carrier? | 24 in 25 (96%) | 7 of 8 (first answer given)  8 of 8 (after self-correction) | 9 of 10 (first answer given) 10 of 10 (after self-correction) |
| If both you and your partner are carriers, what is the chance in each pregnancy of having a child that has CF? | 1 in 4 (25%) | 8 of 8 (first answer given) 8 of 8 (after self-correction) | 9 of 10 (first answer given) 10 of 10 (after self-correction) |
| If you are a carrier and you have a child with someone who has never been tested, what are the chances then that the child will have CF? | less than 1 in 100 (less than 1%) | 5 of 8 (first answer given) 5 of 8 (after self-correction) | 8 of 10 (first answer given) 10 of 10 (after self-correction) |
| If both you and your partner are carriers, what is the chance in each pregnancy of having a healthy child? | 3 in 4 (75%) | 8 of 8 (first answer given) 8 of 8 (after self-correction) | 7 of 10 (first answer given) 9 of 10 (after self-correction) |

Table S4. Summary of main changes to reports made from version 1 to version 2.

| Section | Changes |
| --- | --- |
| Reason for Test | - Black to blue - Bold - Acronym “CF” used |
| About The Test | - Section added to explain inheritance and meaning of “carrier” - Acronym “CF” introduced |
| Results Box | - “Result” to “Your Result” – bold - “gene changes” to “gene alterations” |
| What This Result Means | - “What This Result Means” to “What This Result Means for You” - Explanation of “carrier” removed as covered in About The Test - “single altered Cystic Fibrosis gene” to “one copy of your CFTR genes” [positive reports] - “not a carrier of CF” to “not a known carrier of CF” [positive reports] - “gene changes” to “gene alterations” - “assuming that you are of Northern European origin and that the family relationships stated in the referral are correct” removed [negative reports] - General wording and sentence structure changed to improve explanations, grammar and flow; key risk figures represented with both “X in Y” and as a percentage - Sentence order rearranged to improve comprehension of risk, with some figures removed all together and some risk formats altered |
| Next Steps | - Text bold and presented as bullet points (using dashes) - “…but if you have questions about it, talk to your doctor” added after “You do not need to do anything as a result of this test” - “Take this report with you to any appointments” added [Positive / Partner p.Phe508del] |
| More Information and Support | - “If you don’t have access to the internet, contact the doctor who ordered your test” removed - Phone number for Cystic Fibrosis Trust helpline added |
| Layout / Formatting | - Section headings bold - Separate line for each point - More Information and Support section moved under, rather than adjacent to, Next Steps. |
| For Your Records | - “For Your Records” bold - “If there is anything you do not understand, your doctor will help you to interpret this information” to “The information on this page is for health professionals. It is not essential that patients read this section.” |
| Test Methodology | - Risk of a false positive/negative added - Each reference listed on a new line |
| Full Interpretation | - “gene change” to “gene variant” - “assuming that the family relationships are as stated” removed [negative reports] |
| Layout / Formatting | - Full Interpretation section moved above Test Methodology |

Table S5. Summary of main changes to reports made from version 2 to version 3.

| Section | Changes |
| --- | --- |
| Top Section | - Order of patient details changed to reflect usual laboratory use of information i.e. name followed by date of birth. (also on p.2) - “Copies to” added - Details in “Test carried out by” altered to reflect usual laboratory use of information: - “Name” removed - “Date of Test” replaced with “Date received” and “Date Reported” - “Signature” replaced with “Authorised by” |
| About The Test | - Heading bold |
| Results Box | - “No Cystic Fibrosis gene alterations detected” to “No common cystic fibrosis gene alterations detected” [Negative / Partner p.Phe508del] |
| What This Result Means for You | - “each pregnancy” made bold [positive reports & Negative / Partner p.Phe508del] - “affected with CF” to “have CF” [positive reports & Negative / Partner p.Phe508del] - “someone who is not a known carrier of CF” to “someone who has not been tested for CF” [positive reports] - “(some risk remains as your partner may be a carrier but not know)” added [positive reports] - “No alterations were detected” to “No alterations were detected, so you are not a carrier of any of the alterations we tested for” [negative reports] - “This may require an additional test” added [Negative / Family history] - “This is low, so prenatal testing is not offered” removed [Negative / Partner p.Phe508del] - Some wording changed to improve explanations, grammar and flow. - Some risk figures removed |
| Next Steps | - All text bold - Dashes replaced with bullet points - “we could do a more accurate test” to “we could investigate further” [Negative / Family history] - Alternative options for relatives living in/outside of East Anglia moved to page 2 and replaced with “If your relatives would like to be tested, they should ask their GP about CF carrier testing” |
| More Information and Support | - Phone number for the Regional Genetics Service added - “call” to “phone” - “http://” removed from web addresses - *Note.* By request of the Cystic Fibrosis Trust, the website but not the helpline number will appear in the final version of the reports. |
| Layout / Formatting | - About The Test section moved under, rather than above, Your Result box - Dashed lines between sections removed - Pattern of pale blue shading altered to aid visual separation of sections (Design 2) - Incorrect use of capital letters for “cystic fibrosis” amended throughout |
| For Your Records | - All text in box made bold |
| Full Interpretation | - “i” in “interpretation” capitalised in heading - “assuming that he is of Northern European origin” removed - “John Doe’s CF carrier risk could be determined significantly more accurately if details of the familial CFTR gene variants were known” to “If the specific familial CFTR gene variant were known, we could assess John Doe’s CF carrier risk directly with a targeted test” [Negative / Family history] - Paragraph added about carrier testing and where to send samples |
| Layout / Formatting | - Technical results put in a box |

Table S6. Usability problems uncovered through user testing.

| Round | Severity*^a^* | Issue |
| --- | --- | --- |
|  |  |  |
| 1 | 4 | Population risk presented in a confusing, arguably misleading way which  implied to some participants that the recipient’s carrier risk had been “reduced” from 1 in 25 to 1 in 18 (which was not the case and is not a reduction) |
|  | 3 | Ethnicity/family relationships disclaimer on p. 1 caused consternation |
|  | 3 | Basic result (carrier vs. likely noncarrier status) not always understood |
|  | 3 | Percentage of CFTR gene variants not covered by the test (15%) confused with percentage chance of not being a carrier |
|  | 3 | Reference to “gene changes” misinterpreted (“Can genes change throughout the lifecourse or something? I thought you're kind of born with it or you're not”) |
|  | 2 | Concern about *why* there was residual risk of being a carrier on negative reports |
|  | 2 | No phone numbers provided in “More Information and Support” for individuals without internet access |
|  | 2 | Annoyance at imprecision of the “low” risk of being a carrier on negative reports |
|  | 2 | Several participants felt that “You do not need to do anything as a result of this test” alone left them hanging, requested more information about who to contact with questions |
|  | 1 | ‘Next Steps’ section did not remind recipient to take this report to future appointments with the Clinical Genetics Service |
|  |  |  |
|  | 1 | Various cosmetic features made it unnecessarily difficult to visually distinguish between different sections and between recommended “next steps” |
| 2 | 3 | Ethnicity disclaimer on p. 2 caused consternation |
|  | 3 | For negative reports, concern about the 15% of UK CFTR gene variants not covered by the test that seemed highly disproportionate to risk of being a carrier (less than 0.2%); 15% of CFTR gene variants not covered by the test potentially still being confused with the risk of carrying a pathogenic variant |
|  | 2 | Some confusion from unfortunate coincidence of “1 in 25 (4%)” followed by “1 in 4 (25%)” in immediately following sentence |
|  | 2 | Concern that recipients would want a better explanation of exactly why there is a 1 in 4 chance that any given child of two carriers will have cystic fibrosis |
|  | 2 | Participants did not notice heading indicating that page 2 was primarily for their clinician and did not need to be read and understood by patients |
|  | 2 | More clarity requested on why the report cannot be more definitive |
|  | 2 | Phone number of Regional Genetics Service not provided |
|  | 2 | Confusion about how to interpret “affected with CF” in context of interviewer questions; possibility of confusion since this phrase is used on reports as well |
|  | 1 | Differentiating people living inside vs. outside East Anglia is confusing, as next step for people whose relatives would like to be tested is to discuss with GP in either case |
|  | 1 | Easy to skip over “About the Test” section, as eye is drawn to the result first |
|  | 1 | Table on p. 2 hard to read without borders |
|  | 1 | Various requests related to increasing visual clarity (too busy, too many fonts, dashed lines not necessary, needs more white space, etc.) |
|  |  |  |
| 3 | 2 | Mentioning that results “can be upsetting and difficult to take in” on negative reports confusing |
|  | 2 | Participants did not notice heading indicating that page 2 was primarily for their clinician and did not need to be read and understood by patients |
|  | 2 | More clarity requested on why the report cannot be more definitive |
|  | 2 | Confusion about which ‘doctor’ to return to with questions |
|  | 2 | Some confusion about what CFTR means (though no confusion about the fact that it referred to a gene) |
|  | 1 | Could be made clearer which page is the front |
|  | 1 | “If you plan to have children, CF carrier testing can be offered to your partner before any pregnancy” may be more visible if placed in “Next Steps” section, arguably a more appropriate place for it, rather than current location |
|  | 1 | Requests to further increase separation between different sections |
|  |  |  |

*^a^*We used a severity rating scale borrowed from Rubin (1994)*^b^*, which we adapted to our specific case to make issues easier to classify:

1: “The problem occurs rarely, can be circumvented easily, or is dependent on a standard that is outside the product’s boundaries. Could also be a cosmetic problem.”

2: “The user will be able to use the product, but may have to undertake some moderate effort to get around the problem.” We considered this to include wishes for additional information or clarity that could be satisfied by asking one’s GP.

3: “The user will probably use or attempt to use the product, but will be severely limited in his or her ability to do so.” We considered this to include issues that could leave recipients with a serious misconception.

4: “The user is not able to or will not want to use a particular part of the product because of the way that the product has been designed and implemented.” We had one case of this in Round 1, resulting from an issue that would have been a (3) if not for one user’s strong negative emotional reaction.

*^b^*Rubin J. Handbook of usability testing: how to plan, design and conduct effective tests. New York, NY: John Wiley & Sons, 1994.

Table S7. Examples of how specific usability problems were addressed.

| Issue | Response |
| --- | --- |
| Confusion over whether CF “gene changes” – a phrase introduced as a plain-English alternative to “pathogenic variants” – could occur in the future (“What does it mean by no cystic fibrosis gene changes detected? Can genes change throughout the life course or something? I thought you’re kind of born with it or you're not.”) | “Alterations” employed as plain-English alternative to “changes” or “variants” |
| Information about the 85% of pathogenic variants covered by the panel was confused with the risk of being a carrier (e.g. “There’s a 15% chance that you’re still a carrier… I could be in that 15%.”) | Restated for clarification; ultimately removed 85% statistic from patient-facing page altogether as the restatement did not solve the issue |
| Confusion around the juxtaposition of a sentence stating that the risk is now known to be lower than their *a priori* risk (“The fact that we did not detect any changes reduces the chance that you carry a CF gene change slightly, to 1 in 18”) and an unrelated sentence earlier in the report stating the population risk (“In the UK population, around 1 in 25 people are carriers of CF”); this was interpreted as a risk of 1 in 25 that had been “reduced” to a (greater) risk of 1 in 18. | Sentences rephrased for clarity. The further confusion caused by comparing two risks of differing denominators (given that the risk with the larger denominator was misinterpreted as the higher risk in at least one case) was addressed by expressing carrier risk as a percentage and as “1 in 18,” and the *a priori* risk as a percentage only |
| Confusion from unfortunate coincidence of “1 in 25 (4%)” followed by “1 in 4 (25%)” in immediately following sentence | Moved the statement about the 4% of individuals who are carriers of CF in the UK population to a more contextually appropriate place later in the report |
|  |  |

Table S8. Performance of user-centered reports vs. standard reports, summarized. The user-centered report is deemed “better” or “worse” when differences are statistically significant (adjusted α = .01). All values other than percentages are means.

|  | Performance of user-centered report  (user-centered report vs. standard report) |
| --- | --- |
| **Key objectives** |  |
| Risk probability comprehension scores | **Equivalent** (4.95 vs. 4.94) |
| Subjective comprehension scores |  |
| Subjective understanding | **Better**  (5.74 vs. 4.94) |
| Subjective clarity | **Better**  (5.78 vs. 4.65) |
| Communication efficacy scores | **Better**  (3.11 vs. 2.41) |
| **Exploratory objectives** |  |
| Trust scores | **Equivalent**  (6.23 vs. 5.92) |
| Actionability scores | **Better**  (5.41 vs. 4.37) |
| Risk probability interpretation  (What % of interpretations were clearly wrong*^a^*?) | **Equivalent**  (5% vs. 7% wrong) |
| Visibility of result summary (What % of the time was result summary noticed?) | **Worse**  (73% vs. 92%) |
| Ease of understanding result summary scores | **Better**  (6.05 vs. 5.00) |
| **Primary goal: Key objectives** |  |
| Better performance on at least one;  worse performance on none | **Met** |
| **Secondary goal: All objectives** |  |
| Better performance on at least one;  worse performance on none | **Not met** |

*^a^* “Clearly wrong” interpretations were beliefs that the first child of a couple receiving a positive report would “definitely” or “definitely not” have CF, and beliefs that the first child of a couple receiving a negative report would “definitely” or “likely” have CF.

Table S9. Subgroup analyses (Mann-Whitney U-tests).

|  | | Standard report | |  | User-centered report | |  |  |  |
| --- | --- | --- | --- | --- | --- | --- | --- | --- | --- |
|  | Mean | | SD |  | Mean | SD | U | Cohen’s d | p-value |
| **Participants who did not attend university (n = 83)** |  | |  |  |  |  |  |  |  |
| Risk probability comprehension | 4.61 | | 2.43 |  | 4.29 | 2.53 | 872 | -.13 | .72 |
| Subjective understanding | 4.78 | | 1.26 |  | 5.50 | 1.26 | 563 | .57 | .01 |
| Subjective clarity | 4.61 | | 1.32 |  | 5.91 | 1.26 | 382 | 1.0 | <.001 |
| Communication efficacy | 2.43 | | 0.65 |  | 3.10 | 0.57 | 354 | 1.1 | <.001 |
| Actionability | 4.41 | | 1.33 |  | 5.23 | 1.29 | 554 | .62 | .01 |
| Ease of understanding result summary | 4.92 | | 1.63 |  | 5.97 | 1.34 | 514 | .69 | .002 |
| **Participants with low subjective numeracy*^a^* (n = 80)** |  | |  |  |  |  |  |  |  |
| Risk probability comprehension | 3.90 | | 2.36 |  | 3.62 | 2.46 | 853 | -.12 | .60 |
| Subjective comprehension | 4.63 | | 1.16 |  | 5.28 | 1.36 | 538 | .52 | .01 |
| Subjective clarity | 4.46 | | 1.14 |  | 5.49 | 1.41 | 411 | .80 | <.001 |
| Communication efficacy | 2.29 | | 0.69 |  | 2.99 | 0.69 | 368 | 1.0 | <.001 |
| Actionability | 3.97 | | 1.35 |  | 5.17 | 1.25 | 420 | .93 | <.001 |
| Ease of understanding result summary | 4.68 | | 1.57 |  | 5.79 | 1.47 | 470 | .73 | <.001 |
| **Men (n = 55)** |  | |  |  |  |  |  |  |  |
| Risk probability comprehension | 4.63 | | 2.40 |  | 5.29 | 2.23 | 304 | .28 | .20 |
| Subjective comprehension | 4.48 | | 1.34 |  | 5.96 | 1.04 | 141 | 1.2 | <.001 |
| Subjective clarity | 4.26 | | 1.40 |  | 5.96 | 1.04 | 122 | 1.4 | <.001 |
| Communication efficacy | 2.38 | | 0.64 |  | 3.14 | 0.51 | 132 | 1.3 | <.001 |
| Actionability | 4.28 | | 1.40 |  | 5.56 | 1.18 | 184 | .99 | .001 |
| Ease of understanding result summary | 4.81 | | 1.84 |  | 6.43 | 1.03 | 184 | 1.1 | <.001 |
| **Women (n = 134)** |  | |  |  |  |  |  |  |  |
| Risk probability comprehension | 5.00 | | 2.28 |  | 4.87 | 2.34 | 2316 | -.06 | .75 |
| Subjective comprehension | 5.15 | | 1.13 |  | 5.66 | 1.23 | 1645 | .43 | .006 |
| Subjective clarity | 4.81 | | 1.27 |  | 5.72 | 1.25 | 1282 | .72 | <.001 |
| Communication efficacy | 2.43 | | 0.73 |  | 3.13 | 0.56 | 1010 | 1.08 | <.001 |
| Actionability | 4.42 | | 1.51 |  | 5.35 | 1.23 | 1412 | .67 | <.001 |
| Ease of understanding result summary | 5.01 | | 1.58 |  | 5.96 | 1.39 | 1420 | .63 | <.001 |

*^a^* Defined as falling below the 50^th^ percentile of Zikmund-Fisher et al.’s nationally representative sample of U.S. participants. Zikmund-Fisher BJ, Smith DM, Ubel PA, Fagerlin A. Validation of the subjective numeracy scale: Effects of low numeracy on comprehension of risk communications and utility elicitations. *Medical Decision Making* 2007 Sep; 27(5):663-71. In our data, subjective numeracy was only mildly correlated with risk probability comprehension (r = .4) and had significant but even weaker correlations with other variables.
